# Targeting Cancer Metabolism Plasticity with JX06 Nanoparticles via Inhibiting PDK1 Combined with Metformin for Endometrial Cancer Patients with Diabetes

**DOI:** 10.1002/advs.202104472

**Published:** 2022-01-22

**Authors:** Xiao Yang, Yuan Cheng, Jingyi Zhou, Lingpu Zhang, Xingchen Li, Zhiqi Wang, Shenyi Yin, LiRong Zhai, Ting Huang, Xiaotong Wu, Boqiang Shen, Yangyang Dong, Lijun Zhao, Yujing Chi, Yuanyuan Jia, Jiaqi Wang, Yijiao He, Xiying Dong, Haihua Xiao, Jianliu Wang

**Affiliations:** ^1^ Department of Obstetrics and Gynecology Peking University People's Hospital No. 11, Xizhimen South Street, Xicheng District Beijing 100044 China; ^2^ Beijing National Laboratory for Molecular Sciences State Key Laboratory of Polymer Physics and Chemistry Institute of Chemistry Chinese Academy of Sciences Beijing 100190 China; ^3^ College of Future Technology Peking University Beijing 100871 China; ^4^ Department of Central Laboratory & Institute of Clinical Molecular Biology Peking University People's Hospital Beijing 100044 China; ^5^ Peking Union Medical College Hospital Peking Union Medical College and Chinese Academy of Medical Sciences Beijing 100730 China

**Keywords:** cell metabolic reprogramming, diabetes, endometrial cancer, high glucose, metformin, PDK1

## Abstract

Diabetes is closely related to the occurrence of endometrial cancer (EC) and its poor prognosis. However, there is no effective clinical treatment for EC patients with diabetes (patient^EC+/dia+^). To explore new therapeutic targets, Ishikawa is cultured with high glucose (Ishikawa^HG^) mimicking hyperglycemia in patient^EC+/dia+^. Subsequently, it is discovered that Ishikawa^HG^ exhibits glucose metabolic reprogramming characterized by increased glycolysis and decreased oxidative phosphorylation. Further, pyruvate dehydrogenase kinase 1 (PDK1) is identified to promote glycolysis of Ishikawa^HG^ by proteomics. Most importantly, JX06, a novel PDK1 inhibitor combined metformin (Met) significantly inhibits Ishikawa^HG^ proliferation though Ishikawa^HG^ is resistant to Met. Furthermore, a reduction‐sensitive biodegradable polymer is adopted to encapsulate JX06 to form nanoparticles (JX06‐NPs) for drug delivery. It is found that in vitro JX06‐NPs have better inhibitory effect on the growth of Ishikawa^HG^ as well as patient‐derived EC cells (PDC) than JX06. Additionally, it is found that JX06‐NPs can accumulate to the tumor of EC‐bearing mouse with diabetes (mice^EC+/dia+^) after intravenous injection, and JX06‐NPs combined Met can significantly inhibit tumor growth of mice^EC+/dia+^. Taken together, the study demonstrates that the combination of JX06‐NPs and Met can target the cancer metabolism plasticity, which significantly inhibits the growth of EC, thereby provides a new adjuvant therapy for patients^EC+/dia+^.

## Graphical abstract

Diabetes is closely related to the occurrence of endometrial cancer (EC) and its poor prognosis. However, there is no effective clinical treatment for EC patients with diabetes (patient^EC+/dia+^). This study provides a new adjuvant treatment for patients^EC+/dia+^ in clinical practice, suggesting that JX06 to form nanoparticles (JX06‐NPs) combined with metformin is effective for achieving better antitumor effect by targeting cancer metabolism.

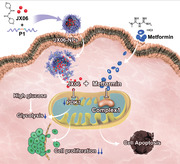

## 1 Introduction

Endometrial cancer (EC) is one of the most common gynecological malignancies. Its pathogenesis is closely related to estrogen imbalance and metabolic disorder. With the increasing incidence of metabolic diseases in the recent years, the incidence and mortality of patients with EC have accelerated globally.^[^ ^1^, ^2^, ^3^ ^]^ Studies have shown that diabetes is a relatively high risk factor for EC. Compared to nondiabetic patients, diabetic patients have twofold increased risk of developing EC. In addition, the mortality of EC patients with diabetes (patients^EC+/dia+^) increases by 41%.^[^ ^4^, ^5^, ^6^, ^7^ ^]^ Taking together, diabetes is important in the pathogenesis and prognosis of EC. However, in current clinical practice, there is no standard treatment for patients^EC+/dia+^. Hyperglycemia is a major clinical feature of diabetes, which is also considered a key link between diabetes and cancer.^[^ ^8^, ^9^ ^]^ Therefore, to control blood glucose level or to interfere with the molecular signaling pathway regarding glucose metabolism may be a new perspective for the clinical treatment of patients^EC+/dia+^.

Metformin (Met) is one of the most common diabetic drugs in the clinic. In recent years, studies have found that it could inhibit the growth of breast cancer, colon cancer, and EC cells. Its main mechanism as an antitumor drug is to inhibit oxidative phosphorylation by blocking the electron transfer of complex I in the mitochondrial respiratory chain and inhibiting the process of oxidative phosphorylation.^[^ ^10^, ^11^, ^12^ ^]^ In addition, a clinical study has shown that Met adjuvant therapy is effective in reversing endometrial atypical hyperplasia, reducing the expression of tumor markers for proliferation, and improving the overall survival of EC patients.^[^ ^13^ ^]^ However, other studies have demonstrated that the inhibitory effect of Met on the growth of EC cells is less prominent under high‐glucose conditions versus that in normal‐glucose conditions,^[^ ^14^ ^]^ which indicates that hyperglycemia may lead to Met resistance in EC cells. As elucidated by the Warburg effect, the glucose metabolism of tumor cells is glycolysis dominant (80%), with oxidative phosphorylation only taking up the rest 20%. In other words, tumor cells may manifest a “metabolic plasticity” or “metabolic reprogramming.”^[^ ^15^, ^16^ ^]^ Further, the mechanism of resistance to Met under hyperglycemic conditions may be related to increased glycolysis.^[^ ^17^ ^]^ Therefore, a combination of glycolytic inhibitors and Met may have enhanced antitumor effect than with Met alone.^[^ ^18^, ^19^ ^]^ The combination of hexokinase 2 (HK2) ablation and Met were proved to be synergistic in inhibiting hepatocellular carcinoma growth.^[^ ^20^ ^]^ However, the role of Met combined with glycolysis inhibitors in EC treatment with comorbidity of diabetes has not been reported. Thus, we hypothesize here that targeting the cancer metabolism plasticity via glycolysis inhibitors combined with Met may increase the sensitivity of EC cells to Met in a high glucose condition, which is of great clinical value for treatments of patients^EC+/dia+^.

To prove this, we first established an EC cell line Ishikawa cultured at a long‐term high glucose (Ishikawa^HG^) concentration, with the same cell line cultured at a normal glucose (Ishikawa^NG^) concentration as the control group. The main reason for choosing a long‐term high‐glucose cell culture was that most previous work had only studied the biological behaviors of EC cells under various glucose concentrations in a short timespan (0–72 h).^[^ ^21^ ^]^ However, diabetes is a chronic metabolic disease, manifesting as long‐term persistent hyperglycemia. Thus, a long‐term culture of cells in a hyperglycemic environment can more accurately simulate the actual in vivo interplay between glucose and EC. Subsequently, with Ishikawa^HG^ and Ishikawa^NG^, we found that 1) compared to Ishikawa^NG^, we observed on Ishikawa^HG^ a hyperglycemic environment exacerbated the transformation of glucose metabolism from oxidative phosphorylation to glycolysis. 2) The expression of pyruvate dehydrogenase kinase 1 (PDK1) for Ishikawa^HG^, a rate‐limiting enzyme of glycolysis, increased significantly as compared to Ishikawa^NG^. Downregulating the expression of PDK1 by short hairpin RNA (shRNA) could remarkably inhibit not only the proliferation, invasion, and glycolysis, but also the AKT/GSK3*β*/*β*‐catenin signaling pathway of Ishikawa^HG^. 3) JX06, a small molecule inhibitor of PDK1, when combined with Met, accelerated the apoptosis of Ishikawa^HG^. Although JX06 is potent in inhibiting PDK1, it has limited solubility in water, fast blood clearance, making it hard to be targeted delivered to the tumor sites and finally enter the cancer cells. Therefore, here we first adopted a reduction‐sensitive polymer (P1) with numerous disulfide bonds in its polymer chain, which could be triggered for degradation by glutathione (GSH)[rr]. Second, JX06 was encapsulated by P1 to form nanoparticles (JX06‐NPs). To fully simulate the pathophysiological characteristics of patient^EC+/dia+^, we subsequently induced diabetes in mice by intraperitoneal injection of streptozocin (STZ), followed by subcutaneous injection of EC cells. At last, we injected JX06‐NPs intravenously into the diabetic EC mice (mice^EC+/dia+^). JX06‐NPs entered cancer cells via the blood circulation, and then accumulated in the tumor sites, dissociated to release JX06 to inhibit PDK1. Met was concurrently administered orally, which played two roles. One was to reduce and control glucose level; the other was to inhibit mitochondrial complex I, suppressing the oxidative phosphorylation of tumor cells. By combining JX06‐NPs and Met, there was an increase of apoptosis of EC cells. Therefore, the combination of JX06‐NPs and Met has shown an enhanced antitumor effect under a high‐glucose condition, providing a new strategy for the treatment of patients^EC+/dia+^.

## 2 Results and Discussion

### 2.1 High Glucose Promoted EC Cell Growth and Reprogramming of Glucose Metabolism

**Figure 1:**
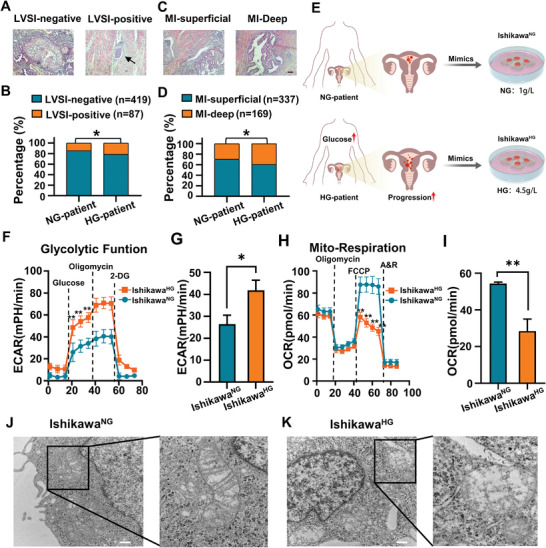
Hyperglycemia promotes the invasion and progression of EC patients via reprogramming of glucose metabolism. A–D) Correlation analysis between hyperglycemia and the incidence of LVSI and MI in EC patients. NG‐patient: LVSI negative (*n* = 261), LVSI positive (*n* = 44); MI‐superficial (*n* = 215), MI‐deep (*n* = 90); HG‐patient: LVSI‐negative (*n* = 158), LVSI‐positive (*n* = 43); MI‐superficial (*n* = 122), MI‐deep (*n* = 79); *P < 0.05. Scale bar: 100 µm. E) The Ishikawa^NG^ and Ishikawa^HG^ were cultured for at least 8 weeks in glucose concentrations of 1 and 4.5 g L^−1^ respectively. F,G) Seahorse energy metabolism analysis was used to detect the effect of high glucose on ECAR. The measured ECAR after the addition of glucose, oligomycin, and 2‐deoxyglucose (2‐DG) represented the glycolytic capacity of cells, the maximum glycolytic capacity, and the mechanisms of acid production other than glycolysis, respectively. H,I) Seahorse energy metabolism analysis was used to detect the effect of high glucose on the OCR of EC cells. The OCR value before adding oligomycin represented the basic oxygen consumption, and FCCP represented the maximum oxygen consumption capacity of mitochondria after uncoupling. J,K) The effect of high glucose on the shape of mitochondria in Ishikawa^HG^ was observed by TEM. The black box marked the location of mitochondria.Scale bar: 500 nm. Data are shown as mean ± standard deviation (SD). B,D) *χ* ^2^ test. F,H) Unpaired Student's *t*‐test. *P<0.05, **P<0.01.

To investigate the relationship between high glucose level and clinicopathological features of EC, the clinic data of 506 patients with EC from the Department of Obstetrics and Gynecology, People's Hospital of Peking University, were analyzed. The results showed that there was no significant correlation between serum glucose levels and tumor grade and stages, lymph node metastasis, or tumor cytology of ascites (*P* > 0.05). However, the proportion of patients with lymph vascular space invasion (LVSI) positive and deep myometrial infiltration (MI‐deep) were significantly higher in patients with high glucose level (HG‐patient) than that in patients with normal glucose level (NG‐patient) (21.39% vs 14.43%; 39.30% vs 29.51%; *P*<0.05) (**Figure** **1**A–D). Baseline characteristics of patients are shown in Table S1 (Supporting Information). LVSI positive and MI‐deep have been reported to be important predictors of lymph node metastasis and poor prognosis of patients.^[^ ^22^, ^23^ ^]^ Therefore, our studies suggested that hyperglycemia was associated with the invasion and progression of EC.

To mimic in vivo normoglycemia and hyperglycemia, Ishikawa^NG^ was cultured in a normal glucose (1 g L^−1^, 5.5 × 10^−3^ m) medium, and Ishikawa^HG^ in a high glucose (4.5 g L^−1^, 25 × 10^−3^ m) medium for at least 8 weeks, and the cells were then used for subsequent experiments (Figure 1E). To study the growth of EC cells, colony formation assay, Transwell invasion assay, and wound healing assay were performed. The results showed that the number of colony formation and invading Ishikawa^HG^ were 1.4 and 1.9 times that of Ishikawa^NG^, respectively. Moreover, the scratch area was decreased for Ishikawa^HG^ versus Ishikawa^NG^ at 24 h (*P*<0.05; Figure S1A–F, Supporting Information). E‐cadherin is a typical marker for epithelial cells, whose decreased expression has been considered a key event during epithelial mesenchymal transition (EMT), the main cause of tumor metastasis.^[^ ^24^, ^25^ ^]^ Therefore, E‐cadherin was marked by immunofluorescence assay, and the mean green fluorescence intensity of E‐cadherin on the membrane of Ishikawa^HG^ was 45% of Ishikawa^NG^ (Figure S1G,H, Supporting Information). The above results proved that a hyperglycemic environment promoted the proliferation, invasion, and metastasis of EC cells.

To further study the effect of high glucose on the glucose metabolism of EC cells, the real‐time changes of oxygen consumption rate (OCR) and extracellular acidification rate (ECAR), which reflected the oxidative phosphorylation and glycolysis energy metabolism, respectively, were assessed via the seahorse XF cell energy metabolism assay.^[^ ^26^, ^27^ ^]^ The results indicated that the glycolysis in Ishikawa^HG^ (ECAR average is 42 mph min^−1^) was significantly much higher than that in Ishikawa^NG^ (ECAR average is 26 mph min^−1^) (Figure 1F,G). Moreover, by adding carbonyl cyanide 4‐(trifluoromethoxy)phenylhydrazone (FCCP), a potent uncoupler of mitochondrial oxidative phosphorylation, the oxygen consumption increased to a maximum. The maximum capacity of mitochondrial respiration in Ishikawa^HG^ was significantly lower than that of Ishikawa^NG^ (OCR: 28 vs 54 pmol min^−1^) (Figure 1H,I). These results suggested that high glucose could trigger a switch to glycolysis and inhibit oxidative phosphorylation.

Mitochondrion is the energy metabolism factory. Studies have shown that the abnormal activation of glycolytic pathway in tumor cells may be closely related to mitochondrial damage.^[^ ^28^, ^29^ ^]^ To further clarify whether mitochondria were affected as hyperglycemia shifts cellular metabolism to more of glycolysis, we used transmission electron microscopy (TEM) to histologically examine the Ishikawa cell lines and found that the mitochondria of Ishikawa^NG^ were morphologically regular; while mitochondria of Ishikawa^HG^ were significantly swollen, with dissolved, broken, or disappeared cristae (Figure 1J,K). To better observe the structural changes of mitochondria, the cells were labeled with mitochondrial probes, and their morphology of mitochondria was examined under a confocal microscope. Results showed that the mitochondria were fragmentated, and the number of rod‐shaped mitochondria was reduced (Figure S1I, Supporting Information), which was consistent with that by TEM. The studies above demonstrated that mitochondrial damage induced by high glucose levels could partially explain the observed reprogramming of cellular metabolism to a glycolysis‐dominant one in EC cells under a hyperglycemic condition. This shift in the mode of metabolism could be a key reason for the progression to malignancy and the poor prognosis of EC.

### 2.2 High Glucose Triggered the Shift in Glucose Metabolism by Promoting the Expression of PDK1

**Figure 2:**
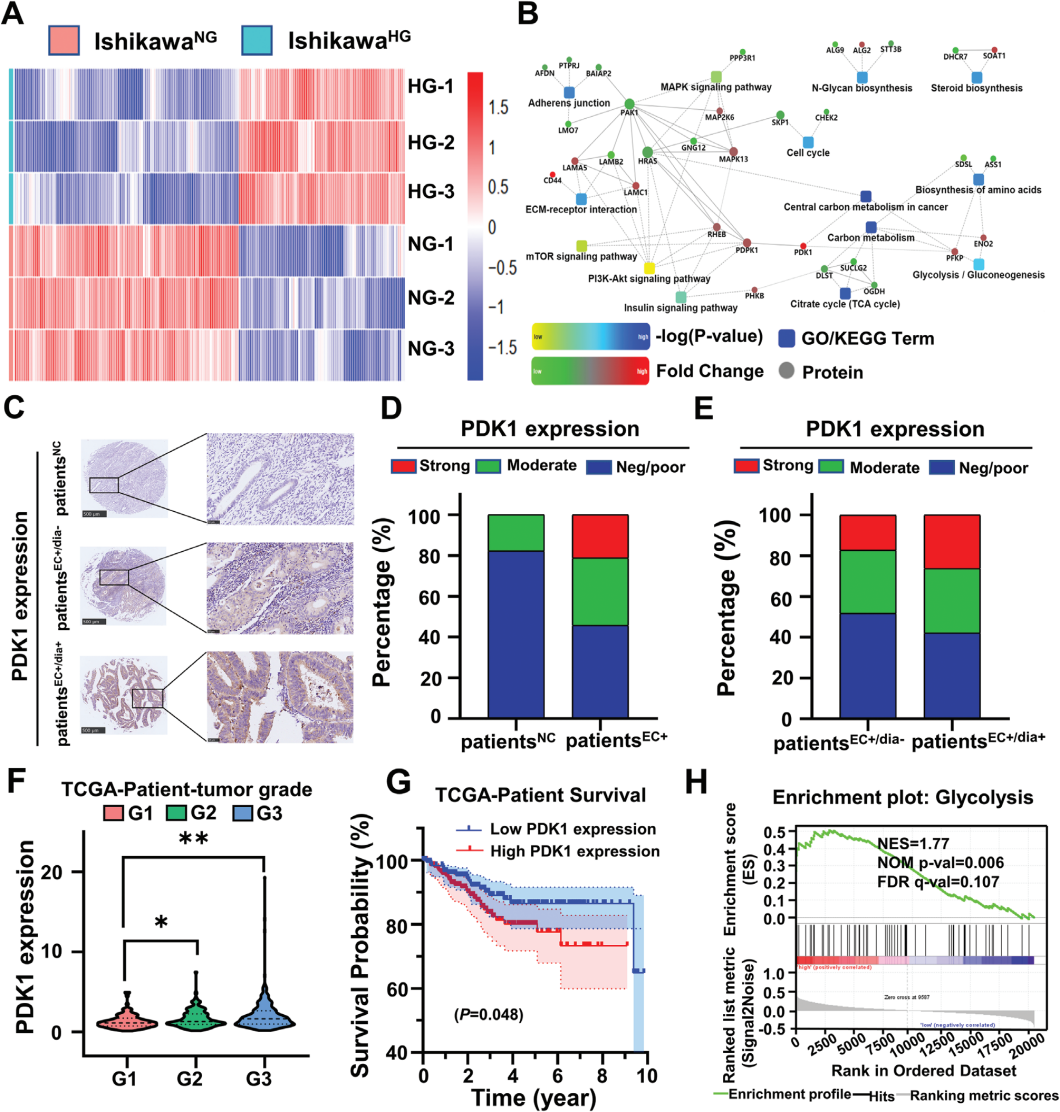
Screening and validation of the differentially expressed proteins between Ishikawa^HG^ and Ishikawa^NG^. A) Hierarchical clustering heat map of differentially expressed proteins in Ishikawa^HG^ versus Ishikawa^NG^ via label‐free mass spectrometry proteomics. Each column in the map represents a protein, and each row represents a group of samples. Red represents the upregulated proteins, blue represents the downregulated proteins, and white represents no quantitative information on that protein. B) Analysis of the protein–protein interaction of the differentially expressed proteins in Ishikawa^HG^ and Ishikawa^NG^. The square in the figure represents the GO/KEGG signaling pathway; the circle represents protein; the line represents the interaction between proteins and pathways; the red circle represents the upregulated proteins; and the green circle represents the downregulated proteins. C) The expression of PDK1 in EC and normal adjacent normal tissues was detected by immuno‐histochemistry. Yellow and brown were positive stains for PDK1. Adjacent normal control endometrial tissue samples (patient^NC^, *n* = 17), EC tissue samples (patient^EC+^, *n* = 118), nondiabetic EC (patients^EC+/dia−^, *n* = 116) and diabetic EC (patients^EC+/dia+^, *n* = 19). Scale bar: 500 µm. The scale bar of the enlarged drawing is 50 µm. D,E) are the statistical results of panels (C). F) The relationship between PDK1 expression in EC tissues and tumor grades in the TCGA database (*n* = 543). One‐way analysis of variance (ANOVA). G) According to the median value, the degree of PDK1 expression in type I EC in TCGA database were divided into either low levels (*n* = 198) or high levels (*n* = 197). Survival curves of EC patients with high (*n*  =  197) and low (*n*  =  198) PDK1 expression, *P* = 0.048. Kaplan–Meier. H) GSEA analysis of the signaling pathway in which PDK1 was expressed in significantly high levels. Data are shown as mean ± SD. *P < 0.05, **P < 0.01.

The aforementioned studies revealed that high glucose could reprogram glucose metabolism in EC cells, but the specific molecular pathway behind this phenomenon was not clear. Therefore, label‐free mass spectrometry proteomics was first used to screen the differentially expressed proteins in Ishikawa^HG^ and Ishikawa^NG^ to see the difference between the two (Figure S2A–D, Supporting Information). Results demonstrated that a total of 216 proteins, from Ishikawa^HG^, showed significant alterations compared with Ishikawa^NG^, of which 92 were upregulated and 124 were downregulated (**Figure** **2**A; Figure S2E, Supporting Information). In addition, Gene Ontology (GO) and Kyoto Encyclopedia of Genes and Genomes (KEGG) enrichment analyses indicated that those differentially expressed proteins were associated with pyruvate metabolism, adherent junction, citric acid cycle (Tricarboxylic Acid Cycle, TCA cycle), and other cancer‐related signaling pathways (Figure S3, Supporting Information). Subsequently, the protein–protein interaction of the differentially expressed proteins was analyzed to further filter out the pivotal ones involved in the hyperglycemia‐related EC progression. It was found that several catalytic enzymes (DLST, SUCLG2, and OGDH) from the *α*‐ketoglutarate to succinyl‐CoA pathway in the TCA cycle were significantly downregulated, indicating that a hyperglycemic condition inhibited the TCA cycle in Ishikawa cells. In particular, PDK1, phosphofructokinase platelet (PFKP), and enolase 2 (ENO2) from the glycolytic pathway were significantly upregulated (Figure 2B). Previous studies have shown that PDK1 was the key regulatory enzyme for the metabolic switch from oxidative phosphorylation to glycolysis by phosphorylating pyruvate dehydrogenase (PDH) to its inactive form, p‐PDH (ser293), to enhance glycolysis. In addition, PDK1 has been found to be overexpressed in a variety of tumors.^[^ ^30^, ^31^, ^32^, ^33^ ^]^ Notably, PDK1 was one of the top ten most upregulated proteins in Ishikawa^HG^ compared to Ishikawa^NG^ by proteomic analysis. Moreover, PDK1 expression in Ishikawa^HG^ was 3.33 times higher than that in Ishikawa^NG^ (Table S2, Supporting Information). Western blot (WB) and reverse transcription polymerase chain reaction (RT‐PCR) further confirmed that high glucose promoted the expression of PDK1 at both messenger RNA (mRNA) and protein levels (Figure S4A–C, Supporting Information). These results together suggested that high glucose could promote glucose metabolic reprogramming by promoting the expression of PDK1.

To further clarify the role of PDK1 in the development of EC and the effect of high glucose on PDK1 expression, the expression of PDK1 in 118 EC tissue samples (patient^EC+^) and 17 adjacent normal control endometrial tissue samples (patient^NC^) adjacent to EC specimens from EC tissue arrays were studied by immuno‐histochemistry. The samples were divided into three groups based on the level of PDK1 expression: negative/poor positive, moderately positive, and strongly positive. The results indicated that PDK1 was mainly expressed in the cytoplasm of cancer cells (Figure 2C). Moreover, the proportion of tissue samples stained moderately and strongly positive for PDK1 were significantly higher in EC specimens than in normal tissues (33.05% vs 17.65% and 21.19% vs 0%, *P*<0.05) (Figure 2D). When the samples were further categorized by whether a patient had diabetes, they could be regrouped into nondiabetic (patients^EC+/dia−^) and diabetic (patients^EC+/dia+^) tissue samples. In this way, we could further show that the proportion of tissue samples stained strongly positive for PDK1 in the patients^EC+/dia+^ group were significantly higher than that in the patients^EC+/dia−^ group (26.3% vs 17.2%, *P* < 0.05) (Figure 2E). Taken together, the above results provided clinical evidence for glucose's potential regulatory role in PDK1 expression. In addition, the EC mRNA expression profile was downloaded from the Cancer Genome Atlas (TCGA) database, including 35 normal tissues and 552 EC tissues. Then, the PDK1 expression from TCGA was analyzed, and results showed it was significantly higher in EC tissues than in normal tissues (Figure S5A, Supporting Information). Further, the PDK1 expression was also positively correlated with the tumor grade (Figure 2F). Moreover, Kaplan–Meier survival analysis showed that the PDK1 expression was negatively correlated with the 10 year survival rate of patients with type I EC (Figure 2G). Besides, gene set enrichment analysis (GSEA) showed that PDK1 had highly clustered expressions in the glycolysis and the cell cycle signaling pathways (Figure 2H; Figure S5B, Supporting Information). To sum up, high glucose induced a reprogramming of glucose metabolism by upregulating PDK1 expression, which could be closely related to the tumor progression and poor prognosis of patients^EC+/dia+^.

To further confirm the role of PDK1 on the regulation of proliferation and invasion of Ishikawa^HG^ and Ishikawa^NG^, shRNA was transfected to downregulate PDK1 expression (Figure S6A,B, Supporting Information). Furthermore, colony‐forming and Transwell assays were studied to show that the number of colony formed and the number of the invading cells Ishikawa^HG^ in the sh‐PDK1 treatment group decreased by 49% and 80%, respectively, compared with the sh‐NC treatment group (*P*<0.01) (Figure S6C‐F, Supporting Information). This result demonstrated that downregulation of PDK1 in Ishikawa^HG^ could significantly inhibit their proliferation and invasion. To further explore the role of PDK1 in the regulation of glycolysis induced by high glucose, ECAR was detected by the seahorse XF cell energy metabolism assay. The results showed that the glycolytic ability of Ishikawa^HG^ from the sh‐PDK1 treatment group was significantly lower than that of the sh‐NC treatment group (ECAR average: 26 vs 46 mPH min^−1^) (Figure S7, Supporting Information). These aforementioned results suggested that Ishikawa^HG^ has increased level of glycolysis, while inhibition of PDK1 could reverse this change in the glucose metabolism.

To verify our results in vivo, we constructed a diabetic BALB/c nude mice model (Mice^Dia+^). Thereafter, Ishikawa^HG^ transfected with sh‐NC or sh‐PDK1 was injected subcutaneously into the Mice^Dia+^. In this way, diabetic EC mouse models of Mice^Dia+/sh‐NC^ and Mice^Dia+/sh‐PDK1^ were obtained. Results indicated that reduced PDK1 expression results in smaller tumor sizes. We can clearly see that the tumor volume in the Mice^Dia+/sh‐PDK1^ was on average 60% that in the Mice^Dia+/sh‐NC^ (Figure S8, Supporting Information). Taken together, the above results showed that downregulation of PDK1 could inhibit the progrowth of EC cells in hyperglycemia.

In addition to directly regulating the glycolysis, PDK1 also participates in a variety of tumor signaling pathways accounting for pathologically malignant behaviors such as proliferation, invasion, and metastasis. Subsequently, to explore the molecular signaling pathway downstream of PDK1, a protein phosphorylation antibody array was adopted to assess the levels of phosphorylated proteins of Ishikawa^HG^ transfected with sh‐NC and sh‐PDK1. Results showed that a total of 20 phosphorylated proteins that had significant different levels of expressions between Ishikawa^HG^ transfected with sh‐NC and sh‐PDK1 were identified. Compared to Ishikawa^HG^ transfected with sh‐NC, 16 proteins were downregulated, and 4 proteins were upregulated Ishikawa^HG^ transfected with sh‐PDK1(Figure S9A,B, Supporting Information). Considering that most differentially expressed phosphorylated proteins went toward a downward trend after the PDK1 knockdown, downregulated proteins were the focus of the following studies. Then KEGG enrichment analysis was performed on the differentially expressed proteins. The results showed that the differentially expressed proteins were clustered in the PI3K–Akt and mechanistic target of rapamycin kinase (mTOR) signaling pathways (Figure S9C, Supporting Information). These pathways have been proven to be closely related to malignant behaviors, such as tumor proliferation, invasion, and metastasis. Further, we noticed that GSK3*β*, downstream of AKT, was among the differentially expressed proteins, and the abnormal activation of AKT/GSK3*β*/*β*‐catenin signaling pathway was involved in the proliferation, invasion, and metastasis of colorectal carcinoma, gastric cancer, and other tumors.^[^ ^34^, ^35^, ^36^, ^37^ ^]^ The regulatory role of high glucose on *β*‐catenin was also reported to be an important mechanism accounting for the association between diabetes and cancer.^[^ ^38^ ^]^ Therefore, we hypothesized that PDK1 could also affect the progression of endometrial carcinoma by regulating AKT/GSK3*β*/*β*‐catenin signaling pathway. To test this hypothesis, western blot was performed. The result showed that compared to Ishikawa^NG^, P‐AKT, P‐GSK3*β*, and *β*‐catenin were significantly upregulated in Ishikawa^HG^, while knocking down PDK1 reversed that upsurge (Figure S9D–G, Supporting Information). These data suggested that HG could promote the proliferation and invasion of Ishikawa cells via the PDK1–AKT/GSK3β/β‐catenin signaling pathway. The meaning has not been changed.

### 2.3 In Vitro Antitumor Effect of JX06‐NPs Combined with Met

According to above study, the key enzyme PDK1 of glycolysis could be a potential therapeutic target for patients^EC+/dia+^. JX06 is an effective, selective, and covalent PDK1 inhibitor with significant antitumor effect. It covalently binds the cysteine residues of PDK1 molecule in an irreversible manner to inhibit its activity.^[^ ^39^ ^]^ To confirm its antitumor activity, the growth rate of Ishikawa^HG^ was studied, and results showed that it decreased in a JX06 concentration‐dependent manner. The half‐maximal inhibition (IC_50_) values of JX06 on Ishikawa^HG^ at 24 and 48 h were ≈0.65× 10^−6^ and ≈0.35 × 10^−6^ m, respectively (Figure S10A, Supporting Information). Similarly, flow cytometry study showed that JX06 induced a higher apoptotic rate in Ishikawa^HG^ versus that of phosphate buffer saline (PBS) treatment group at 48 h (Figure S10B, Supporting Information). In addition, western blot results showed that JX06 treatment on Ishikawa^HG^ could significantly downregulate p‐PDHA1 expression compared to the p‐PDHA1 level observed in the PBS treatment group (Figure S10C, Supporting Information). These results indicated that JX06, as a PDK1 inhibitor, could effectively impede high glucose's growth‐promoting effect on Ishikawa^HG^ cells.

**Figure 3:**
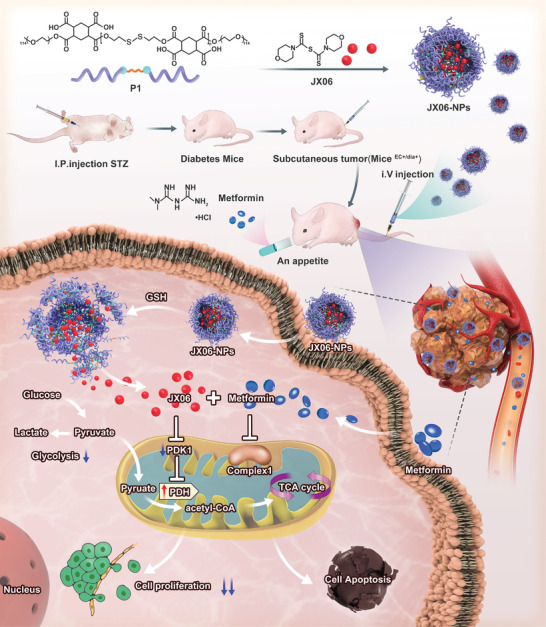
Schematic illustration of JX06‐NPs downregulating the expression of PDK1 combined with Met for targeting cancer plasticity and reprogramming the glucose metabolism for synergistic antitumor effect. A reduction‐sensitive polymer (P1) was adopted to encapsulate JX06 to form JX06‐NPs. JX06‐NPs were then intravenously injected into mice^EC+/dia+^, and Met was administered orally. Met could not only reduce the blood glucose levels, but also inhibit mitochondrial complex I and oxidative phosphorylation. Thereafter, JX06‐NPs could release JX06 after entering cancer cells, downregulating the expression of PDK1, which subsequently inhibits glycolysis. Therefore, JX06‐NPs combined with Met could accelerate the apoptosis of EC and inhibit tumor growth.

**Figure 4:**
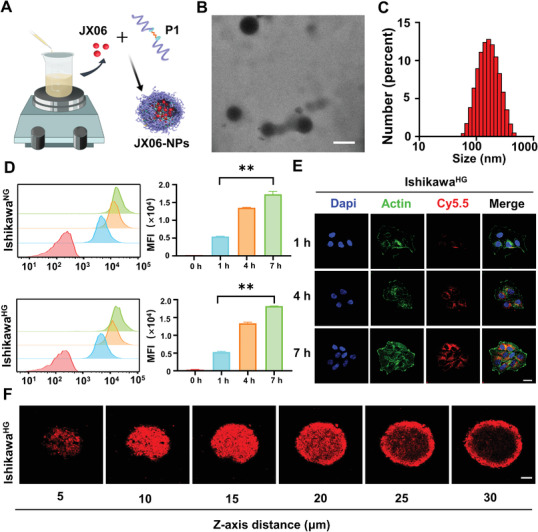
Preparation, characterization, and the cellular uptake of JX06‐NPs. A) Preparation of JX06‐NPs. B,C) Characterization of JX06‐NPs by TEM and DLS. Scale bar: 100 nm. D,E) The intracellular uptake of JX06‐NPs by Ishikawa^HG^ and Ishikawa^NG^ at various time points from 0 to 7 h detected by flow cytometry and CLSM. JX06‐NPs were labeled by Cy5.5. Scale bar: 20 µm. Unpaired Student's t‐test. F) 3D cell spheres of Ishikawa^HG^ treated with JX06‐NPs were observed by CLSM from different sections (5–30 µm). Scale bar: 100 µm. Data are shown as mean ± SD. **P < 0.01.

High glucose could be an important factor accounting for the resistance of tumor cells to Met.^[^ ^14^ ^]^ We found that (5–20) × 10^−3^ m of Met could significantly inhibit the proliferation of both Ishikawa^HG^ and Ishikawa^NG^. However, this inhibitory effect on Ishikawa^HG^ was not as prominent as that observed in Ishikawa^NG^. The inhibition rate of 5 × 10^−3^ m Met was 35% for Ishikawa^NG^ and 30% for Ishikawa^HG^ (*P* < 0.05) (Figure S11A, Supporting Information). This confirmed that Ishikawa^HG^ gained a greater resistance to Met. Met is known to inhibit mitochondrial complex I and block the electron transfer of complex I in the mitochondrial respiratory chain, thus inhibiting the growth of tumor cells in vitro and in vivo.^[^ ^40^ ^]^ A mitochondrial complex I inhibitor rotenone was also used in comparison to metformin in our study. We found that there were no obviously differences as the IC_50._ of rotenone was ≈0.2 × 10^−6^ m both on Ishikawa^NG^ and Ishikawa^HG^ (Figure S11B, Supporting Information). It was reported that the inhibition of Met on mitochondrial complex I was distinct from the complex I inhibitor rotenone. Rotenone is highly toxic, and its accumulation in cells does not require a specific membrane potential, while Met requires a robust mitochondrial membrane potential and can inhibit complex I reversibly.^[^ ^41^ ^]^ The increased glycolysis and oxidative phosphorylation have been proposed as a resistance mechanism of Met. Thus, this resistance could be due to the increased glycolysis of Ishikawa^HG^ compared with Ishikawa^NG^. Therefore, we assumed that JX06 as an inhibitor of PDK1 might work synergistically with Met to exert an anticancer effect. To verify this hypothesis, the anticancer activity of Met combined with JX06 was studied on Ishikawa^HG^, and the results showed that the IC_50_ of Met alone was 13.47 × 10^−3^ m versus 11.39 × 10^−3^ m for Met with 0.4 × 10^−6^ m of JX06 and 8.84 × 10^−3^ m for Met combined with 0.6 × 10^−6^ m of JX06. This indicated that the IC_50_ of Met went on a decreasing trend when combined with JX06 (Figure S11C–E, Supporting Information). Thereafter, the combination index (CI) was calculated to assess the synergistic effect between Met and JX06 by using compuSyn software.^[^ ^42^ ^]^ It showed that the CI values of Met and JX06 on ishikawa cells were calculated to be less than 1 (Figure S11F,G, Supporting Information). To explore the effect of JX06 and Met on the glycolysis levels, we further detected the lactate production after treatment with single and combinational uses of Met and JX06. Our results demonstrated that treatment with JX06 could significantly inhibit the glycolysis since the lactate production was decreased by 0.21 compared with PBS. However, Met resulted into increased lactate production compared with PBS. It may be related to the inhibitory effect of Met on oxidative phosphorylation. In addition, the lactate production in Ishikawa^HG^ was 1.32 times as much as that in Ishikawa^NG^. Compared with Met, the lactate production was decreased in cells treated with Met combined with JX06 (Figure S12, Supporting Information). These results indicated that Met and JX06 could have a synergistic antitumor effect in patients^EC+/dia+^. Nanocarrier is used for targeted delivery due to its advantages, such as its small size, large surface area, good biocompatibility, biodegradability, and controlled release behavior in vivo.^[^ ^43^, ^44^ ^]^ JX06 is a small molecule with limited water solubility. To deliver JX06 in a safer and more efficient way, JX06‐NPs were prepared (**Figures** **3** and **4**A). Figure 4B showed a TME image of JX06‐NPs, exhibiting spherical morphology. Dynamic light scattering (DLS) results showed that the average size of JX06‐NPs was 148.1 nm(Figure 4C). To observe the intracellular uptake of JX06‐NPs by Ishikawa^HG^ cells, JX06‐NPs were further labeled with Cy5.5. Flow cytometry was often used to observe cellular uptake.^[^ ^45^, ^46^ ^]^ Flow cytometry study showed that the uptake of JX06‐NPs increased overtime, and the intracellular fluorescence intensity at the 7 h was three times than at 1 h. Moreover, there was no significant difference in the uptake of JX06‐NPs between Ishikawa^HG^ and Ishikawa^NG^ (Figure 4D). We further observed the internalization of JX06‐NPs in Ishikawa^HG^ by a confocal laser scanning microscope (CLSM) and found that the intensity of red fluorescence in cells increased overtime, suggesting that the uptake of JX06‐NPs by Ishikawa^HG^ gradually increased (Figure 4E). In order to further simulate the tumor microenvironment and the 3D spatial structure of tumor in vivo, an in vitro 3D cell culture spheroid was established, and the uptake of JX06‐NPs at different tumor sections in the 3D sphere was observed by the CLSM. It was found that from 5 to 20 µm of the sphere section, the intensity of red fluorescence increased significantly, while from 20 to 30 µm of the sphere section, the intensity of red fluorescence at the center of the sphere gradually decreased (Figure 4F). JX06 encapsulation efficiency, loading capacity, and drug release were also evaluated. The results showed that JX06 encapsulation efficiency was 40% and the loading capacity was 4%. In addition, the release of JX06 from JX06‐NPs was measured, and the results indicated that there was a gradual increase in drug release at pH 7.4 and GSH =10 × 10^−3^ m from 0 to 24 h. The drug releases were 24.83% (pH = 7.4) and 76.08% (GSH = 10 × 10^−3^ m) at 24 h (Figure S13, Supporting Information). The above results showed that both Ishikawa^HG^ and Ishikawa^NG^ cells were well able to uptake JX06‐NPs.

**Figure 5:**
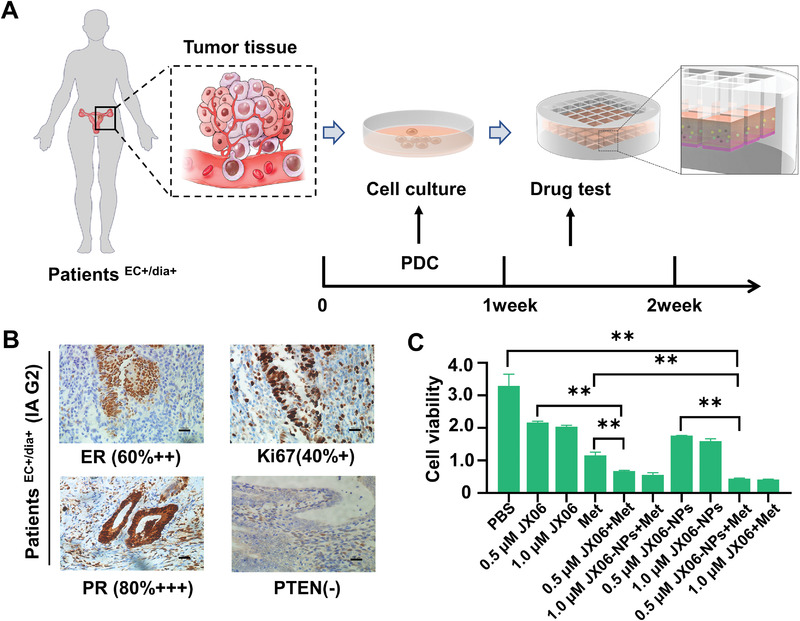
In vitro anticancer activity of JX06‐NPs combined with Met on PDC cells from patient^EC+/dia+^ diagnosed with stage IA grade 2 (IAG2) EC. A) Flowcharts of PDC for the drug‐sensitivity test. B) Tumor specimen was collected from patient^EC+/dia+^ diagnosed with stage IA grade 2 (IAG2) EC, and immuno‐histochemistry staining showed ER (60%++), Ki67 (40%+), PR (80%+++), and phosphatase and tensin homolog (PTEN) (−). C) Viability of PDC cells after treatment with JX06, JX06‐NPs, and Met. Data are shown as mean ± SD. One‐way ANOVA. **P < 0.01.

Patient‐derived cells (PDC) have been used in drug sensitivity tests.^[^ ^47^ ^]^ To more accurately simulate the in vivo conditions, we used PDC from patients^EC+/dia+^ to verify the inhibitory effect of JX06‐NPs and Met. Notably, the above tumor tissues for PDC were collected from patients^EC+/dia+^ diagnosed as stage IA grade 2 (IAG2), and the drug sensitivity test schedule is shown in **Figure** **5**A,B. Subsequently, we found that the inhibitory rate of Met (5 × 10^−3^ m) was 65%, JX06 (0.5 × 10^−6^ m) was 34%, and JX06‐NPs (0.5 × 10^−6^ m) was 46%. This clearly showed that the inhibitory effect of JX06‐NPs was stronger than JX06. In addition, the antitumor effect of either JX06 or JX06‐NPs combined with Met was significantly stronger than the single use of JX06, JX06‐NPs, or Met. Moreover, JX06‐NPs combined with Met had an inhibitory rate at 86% as compared to JX06 combined with Met at 79% (Figure 5C). To further explore the inhibitory effect of JX06‐NPs on Ishikawa^HG^, an apoptosis assay was performed, and the results indicated that compared with PBS, JX06‐NPs could significantly induce an apoptosis rate of 10.66%. The apoptosis rate of Ishikawa^HG^ treated with JX06‐NPs combined with Met (14.57%) was higher than Met alone (2.78%) and JX06‐NPs alone (Figure S14, Supporting Information). In addition to inhibit mitochondrial complex I, Met has also been reported to inhibit the proliferation of cancer cells by inhibiting PI3K/mTOR signaling and glyoxalase 1 (GLO1) expression.^[^ ^48^, ^49^ ^]^ To further study the effect of the single use and combinational use of Met and JX06‐NPs on PI3K/mTOR signaling and GLO1, WB was performed to detect the expression of p70s6k phosphorylation (P‐p70s6K, downstream molecule of mTOR pathway) and GLO1 after drug treatment. The results showed both the expressions of P‐p70s6K and GLO1 were decreased in Ishikawa^HG^ treated with 5 × 10^−3^ or 10 × 10^−3^ m Met compared with PBS (For P‐p70s6K with 5 × 10^−3^ or 10 × 10^−3^ m: 0.34 or 0.38 vs 0.64; for GLO1 with 5 × 10^−3^ or 10 × 10^−3^ m: 0.41 or 0.45 vs 0.59). The combinational use of Met and JX06‐NPs also showed to inhibit the expression of p70s6K and GLO1 compared with PBS, while there was no significant difference compared with single Met (Figure S15, Supporting Information). Since metformin can indirectly inhibit cellular proliferation by blocking growth‐promoting pathways that lie downstream from the insulin receptor,^[^ ^50^ ^]^ the antitumor effect of Met may depend on direct and indirect ways in vivo. These results together suggested that JX06‐NPs combined with Met could have a synergistic antitumor effect.

### 2.4 Combined Antitumor Effect Induced by JX06‐NPs and Met In Vivo

**Figure 6:**
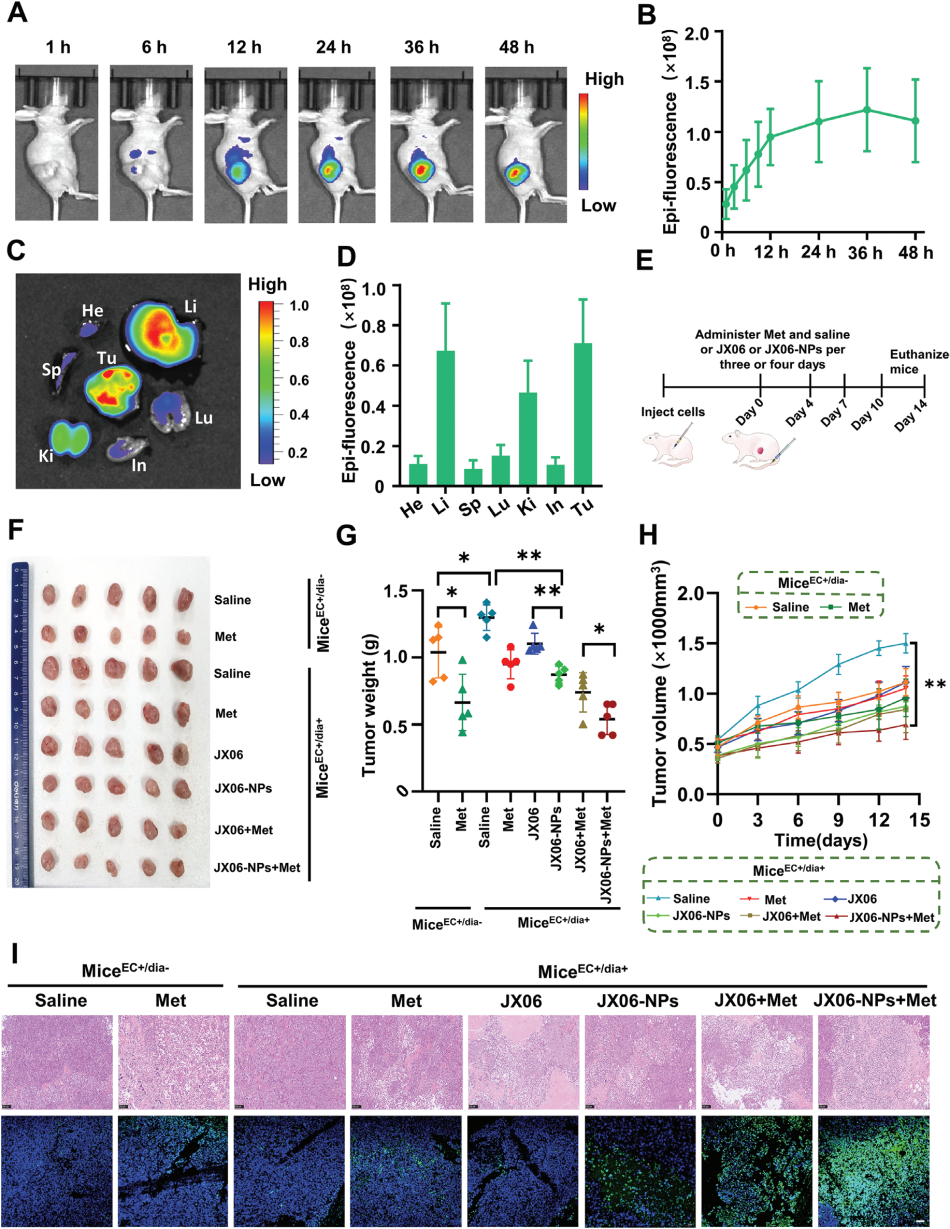
In vivo anticancer activity of JX06‐NPs on a mice^EC+/dia+^ model. A) In vivo fluorescent imaging was performed for the biodistribution of JX06‐NPs. JX06‐NPs were labeled with Cy7.5. B) Semiquantitative study of the fluorescence intensity of JX06‐NPs in tumor tissues at different time points. C) The mice were sacrificed 48 h after JX06‐NPs injection, and the major organs were dissected. The distribution of JX06‐NPs in each organ was observed by fluorescent imaging. D) In vitro distribution of fluorescence in JX06‐NPs in various organs. E) Treatment schedule for efficacy study. F) Images of tumor tissues isolated from mice. G,H) Weight and tumor volume were shown. The data were shown in mean ± standard deviation (*n* = 5). One‐way ANOVA. I) H&E and TUNEL staining were used to detect the changes in cellular structure and the incidence of apoptosis. Data are shown as mean ± SD. *P<0.05, **P<0.01.

Next, we constructed a subcutaneous diabetic EC tumor‐bearing mice model (mice^EC+/dia+^) to evaluate JX06‐NPs combined Met in vivo. First, to observe the accumulation of JX06‐NPs, JX06‐NPs were labeled with Cy7.5 and injected intravenously. The in vivo imaging showed that the fluorescence signal gradually increased at the tumor site overtime and reached to the maximum at 36 h (**Figure** **6**A,B). JX06‐NPs could possibly accumulate into the tumors via enhanced permeability and retention (EPR) effects.^[^ ^51^ ^]^ After 48 h, the mice were killed, and their main organs were further collected for in vitro imaging. The results showed that the fluorescence signal was strong in tumor tissues (Figure 6C). Further fluorescence semiquantitative analysis demonstrated that the relative fluorescence signals were mainly distributed in tumor (7.11 × 10^7^) and liver (6.73 × 10^7^) (Figure 6D). The above results together indicated that JX06‐NPs could effectively target tumor tissues.

Subsequently, to evaluate the therapeutic effect of JX06‐NPs combined with Met in vivo, mice^EC+/dia+^ and nondiabetes EC (mice^EC+/dia−^) models were constructed. The treatment schedule for in vivo study is shown in Figure 6E. JX06 and JX06‐NPs were administered intravenously, and Met was administered orally. In addition, at the end of treatment, the mice were sacrificed and their tumor tissues were collected for photographing (Figure 6F). The tumor weight of mice^EC+/dia+^ treated with saline was 1.3 times greater than that in mice^EC+/dia−^, confirming that high glucose promoted tumor growth. Moreover, Met treatment in mice^EC+/dia+^ and mice^EC+/dia−^ both showed inhibitory effect on tumor weight. The tumor weight in mice^EC+/dia−^ treated with saline was 1.6 times higher than that with Met. However, the tumor weight of mice^EC+/dia+^ treated with saline was 1.4 times greater than that treated with Met. As Met is a widely used diabetic drug, we hypothesized that the tumor inhibition of Met on mice^EC+/dia+^ may be closely related to its hypoglycemic effect. For the nanoparticles’ treatment group, we observed that the tumor weight of mice^EC+/dia+^ treated with JX06‐NPs was 0.872 g, significantly lower than that treated with JX06 (1.102 g) and saline (1.298 g), suggesting that JX06‐NPs could more significantly inhibit EC growth. Moreover, for the combination groups, JX06‐NPs combined with Met have the strongest inhibitory effect on mice^EC+/dia+^, and its antitumor effect is even stronger than that of JX06 combined with Met (average tumor weight: 0.54 vs 0.74 g) (Figure 6G). We could also see from the tumor growth curve and found that JX06‐NPs combined with Met showed the minimal tumor volume (Figure 6H). Finally, hematoxylin‐eosin (H&E) staining analysis showed that tumor tissue in mice^EC+/dia+^ treated with JX06‐NPs had large necrosis, unclear cell structure, and nuclear shrinkage. Further, TdT‐mediated dUTP nick end labeling (TUNEL) staining results showed that the green fluorescence intensity of tumor cells in mice^EC+/dia+^ treated with JX06‐NPs, and Met was significantly increased, indicating more apoptosis of EC cells, which is consistent with H&E staining results (Figure 6I). To further verify the effect of JX06 on PDK1 expression after treatments, we detected the expression of p‐PDHA1[Ser 293] (an indirect indicator of the PDK1 inhibition^[^ ^39^ ^]^) in tumor tissue from mice^EC+/dia+^ after treatment by immuno‐histochemistry. The results showed that compared with mice^EC+/dia+^ treated with saline, the expression of p‐PDHA1 was significantly decreased in mice^EC+/dia+^ treated with JX06, JX06‐NPs, JX06+Met, and JX06‐NPs+Met, since brown granules were decreased after treatments (Figure S16, Supporting Information). The above results together suggested that JX06‐NPs combined with Met had an enhanced antitumor effect.

The animal experiment was approved by the ethics committee of Peking University People's hospital (2020PHE093), and the ethical requirements of experimental animals and the animal welfare law were strictly adhered to during the experimental operations. The human study was approved by the ethics committee of the Peking University People’s Hospital.

## 3 Conclusions

In summary, our study found that high glucose was closely related to LVSI positive and MI‐deep of EC. By establishing cell lines of Ishikawa^HG^ and Ishikawa^NG^, we found that long‐term high‐glucose culture exacerbated the transformation of metabolism from oxidative phosphorylation to glycolysis of EC. Subsequently, through mass spectrometry proteomic screening, we found that the expression of PDK1, a key enzyme of glycolysis in Ishikawa^HG^ was 3.33 times higher than that in Ishikawa^NG^. We further used shRNA to knockdown PDK1 and found it significantly inhibited the proliferation, invasion, glycolysis, and AKT/GSK3*β*/*β*‐catenin signaling pathway of Ishikawa^HG^, which confirmed the role of PDK1 in the malignant biological behavior of EC cells induced by high glucose. Next, we found that the inhibition rate of JX06, a PDK1 inhibitor combined with Met, on Ishikawa^HG^ was 2.5 times higher than that of Met alone. We subsequently delivered JX06 with JX06‐NPs with higher intracellular uptake and greater inhibitory effect. Moreover, the apoptosis rate on Ishikawa^HG^ induced by JX06‐NPs combined with Met was 5.2 times than that of Met alone. More importantly, through the PDC from patients^EC+/dia+^, we found that the growth inhibition rate of JX‐06 NPs combined with Met was 1.32 times higher than that of Met alone, and its mechanism may be related to the inhibition of glycolysis pathway and oxidative phosphorylation dual metabolic pathway. Further, we established mouse models of mice^EC+/dia+^ and mice^EC+/dia−^ and showed that the inhibition rate of Met on mice^EC+/dia+^ was less than that in mice^EC+/dia−^. On the mice^EC+/dia+^ model, JX06‐NPs and Met had combined inhibitory anticancer effects. Our study provides a paradigm of new adjuvant treatment for patients^EC+/dia+^ in the near future clinical practice, suggesting that Met combined with JX06‐NPs is effective for achieving better antitumor effect.

## Conflict of Interest

The authors declare no conflict of interest.

## Author Contributions

X.Y., Y.C., and J.Z. contributed equally to this work. JL.W., H.X., X.Y. contributed to the design of the project and mainly contributed to data analysis and drafting of the manuscript. LP.Z. synthesized the nanopaticles. X.L., LR.Z., T.H., X.W., and B.S. provided assistance in animal experiments. Y.D., Z.W., LJ.Z. provided clinical data. S.Y., Y.J., JQ.W., and Y.H. provided experimental guidance. X.D. helped to polish the language.

## Supporting information

Supporting Information

## Data Availability

The data that support the findings of this study are available from the corresponding author upon reasonable request.
