# Supplementary material for: Targeting Cancer Metabolism Plasticity with JX06 Nanoparticles via Inhibiting PDK1 Combined with Metformin for Endometrial Cancer Patients with Diabetes
Source: Adv Sci (Weinh). 2022 Jan 22;9(8):2104472. doi: 10.1002/advs.202104472 (PMC8922133; doi:10.1002/advs.202104472)
Supplement: Supplementary file 1 — Supporting Information [file ADVS-9-2104472-s001.pdf]

ADVANCED SCIENCE
Open Access

Supporting Information

for *Adv. Sci.*, DOI 10.1002/advs.202104472

Targeting Cancer Metabolism Plasticity with JX06 Nanoparticles via Inhibiting PDK1 Combined with Metformin for Endometrial Cancer Patients with Diabetes

Xiao Yang, Yuan Cheng, Jingyi Zhou, Lingpu Zhang, Xingchen Li, Zhiqi Wang, Shenyi Yin, LiRong Zhai, Ting Huang, Xiaotong Wu, Boqiang Shen, Yangyang Dong, Lijun Zhao, Yujing Chi, Yuanyuan Jia, Jiaqi Wang, Yijiao He, Xiying Dong, Haihua Xiao and Jianliu Wang**

ADVANCED SCIENCE
Open Access

Supporting Information

for *Adv. Sci.*, DOI: 10.1002/advs.202104472

Targeting Cancer Glucose Metabolism Plasticity with JX06 Nanoparticles via Inhibiting PDK1 Combined with Metformin for Endometrial Cancer Patients with Diabetes

Xiao Yang, Yuan Cheng, Jingyi Zhou, Lingpu Zhang, Xingchen Li, Zhiqi Wang, Shenyi Yin, LiRong Zhai, Ting Huang, Xiaotong Wu, Boqiang Shen, Yangyang Dong, Lijun Zhao, Yujing Chi, Yuanyuan Jia, Jiaqi Wang, Yijiao He, Xiying Dong, Haihua Xiao, Jianliu Wang**

Supporting Information

Targeting Cancer Glucose Metabolism Plasticity with JX06 Nanoparticles via Inhibiting PDK1 Combined with Metformin for Endometrial Cancer Patients with Diabetes

Xiao Yang#1, Yuan Cheng#1, Jingyi Zhou#1, Lingpu Zhang2, Xingchen Li1, Zhiqi Wang1, Shenyi Yin3, LiRong Zhai1, Ting Huang1, Xiaotong Wu1, Boqiang Shen1, Yangyang Dong1, Lijun Zhao1, Yujing Chi4, Yuanyuan Jia1, Jiaqi Wang1,Yijiao He1, Xiying Dong5, Haihua Xiao2*, Jianliu Wang1*

1. Department of Obstetrics and Gynecology, Peking University People's Hospital, Beijing, 100044, China

2. Beijing National Laboratory for Molecular Sciences, State Key Laboratory of Polymer Physics and Chemistry, Institute of Chemistry, Chinese Academy of Sciences, Beijing, 100190, China

3. College of Future Technology, Peking University, Beijing 100871, China

4. Department of Central Laboratory & Institute of Clinical Molecular Biology, Peking University People's Hospital, Beijing, 100044, China

5. Peking Union Medical College Hospital, Peking Union Medical College and Chinese Academy of Medical Sciences, Beijing, 100730, China.

#These authors have contributed equally to this work

Corresponding author:

Jianliu Wang. Department of Obstetrics and Gynecology, Peking University People's Hospital, No. 11, Xizhimen South Street, Xicheng District, Beijing 100044, China; Email: wangjianliu@pkuph.edu.cn.

Haihua Xiao. Beijing National Laboratory for Molecular Sciences, State Key Laboratory of Polymer Physics and Chemistry, Institute of Chemistry, Chinese Academy of Sciences, Beijing 100190, China; Email: hhxiao@iccas.ac.cn.

Experimental Section

DMEM medium (with glucose: 1 g/L, CM10013; 4.5 g/L, CM10014), PBS and 0.25% trypsin were purchased from the Macgene company (Beijing, China). Transwell 24-well plates (353097) were purchased from BD Biosciences (China). 96-well Seahorse XFe cell energy metabolism analyzer was used for energy metabolism analyses. All reagents including glucose, 2-DG, oligomycin, Carbonyl cyanide-p-trifluoromethoxyphenylhydrazone (FCCP), antimycinA/rotenone (A&R) were purchased from Sigma. Met were purchased from APExBIO, America; Transmission Electron Microscope was used to document the morphology of mitochondria. Mitobright LT red (MT11) was purchased from Beijing Beiren Chemical Technology Co. Ltd (Beijing, China); The immunohistochemical kit was purchased from Beijing Zhongshan Jinqiao Biotechnology Co. Ltd (Beijing, China). High performance liquid chromatography (HPLC) analysis was performed on Agilent 1200 series instruments. Flow cytometry was done by a flow cytometry analyzer (BD FACScalibur, USA); Fluorescent imaging analysis was performed under a confocal microscope (Leica TCS Sp8, Germany). Anti-PDK1 antibody (3820s), Anti-P-AKT antibody (9271T), Anti-P-GSK3β (9323T), Anti-β-Catenin antibody (8480T) and Anti-E-cadherin antibody (14472s) were purchased from the Cell Signaling Technology (CST); Anti-PDHA1(Phospho S293) (ab177461) and Anti-GLO1 (ab137098) were purchased from abcam company. Lacate Assay Kit (A019-2-1) was purchased from njjcbio company (Naijing, China). Rotenone (B5462) was purchased from APExBIO, USA. Anti-β-Actin antibody (66009-1-IG) was purchased from Proteintech. Goat anti-rabbit IgG (Alexa Fluor® Plus 488) was purchased from Thermofisher, USA. Odyssey infrared fluorescence scanning imaging system was used for the western blot analysis. CCK8 was carried out on infinite M200 multifunctional enzyme-labeling instrument. PCR-related reagents: Trizol reagent were purchased from Invitrogen, and chloroform, isopropanol and absolute ethanol were from Beijing chemical plant; PCR reverse transcription kit was purchased from yeasen biotech Co., Ltd. Western blot related reagents: RIPA protein lysate,

phosphoprotease inhibitor, 5 × Protein loading buffer were purchased from Applygen company, Beijing. EC tissue arrays (EMC1351) were purchased from Superbiotek, Shanghai, China. Annexin V-FITC apoptosis kit was purchased from BD biosciences. AAH-PPP-1 kit was purchased from Ray Biotech, China. Negative control (NC) shRNA sequence and three target gene shRNA sequences (shRNA1 sequence, shRNA2 sequence and shRNA3sequence) were designed for PDK1, and lentivirus packaging was carried out by Hanheng Biotechnology Co., Ltd. The details on sequences are shown in table S3, Supporting Information. Streptozocin (STZ) was purchased from Meilunbio, China.

Cell culture

The Ishikawa cell line was preserved in the Department of Obstetrics and Gynecology of the People's Hospital of Peking University (Beijing, China). $Ishikawa^{NG}$ and $Ishikawa^{HG}$ were cultured in DMEM mediums (glucose 1 g/L and 4.5 g/L) containing 10% FBS respectively and cultured in a 37 ℃ incubator with 5% CO_2. When the degree of cell fusion reached 80 ~ 90%, the cells were digested with 0.25% trypsin, and then sub-cultured or inoculated in cell plates for subsequent experiments.

Seahorse XF energy metabolism analysis

A 200 μl hydration solution was added to each well of the plate below the probe, and the plate was placed in a CO_2-free incubator at 37 ℃ overnight. Take out the cell culture plate (XF96 cell culture microplates), digest the cells with trypsin, and prepare a cellular suspension. The suspension was diluted to the expected concentration of 1.25×10^5 /ml, and 80 μl of which was added to each well of the culture plate. After inoculation, the cells were sat at room temperature for 1 h, then transferred to an incubator to culture, and test was performed after 16-24 h. Glycolytic stress detection medium (50 ml = 49 ml basic medium + 1ml glutamine, pH adjusted to 7.35 ± 0.05) and mitochondrial stress detection medium (normal glucose 55 μl/ high concentration glucose 250 μl+500 μl glutamine+500 μl sodium pyruvate, basic medium to final

volume 25ml) were detected. The cells were washed with medium three times, and 175 µl of fresh medium was added per well. Then, the cell plate was placed in a CO_2 free incubator at 37 ℃ for 1 h. The liquid adding plates were placed (A, B, C and D) on the probe plate respectively, and 25µl of corresponding drugs was added per well. a. Loading sequence of the glycolytic stress detection experiment A: glucose; B: oligomycin; C: 2-DG; b. Loading sequence of the mitochondrial stress detection experiment: A: oligomycin; B: FCCP; C: Antimycin A/Rotenone(A&R). The correction probe plate was set according to the procedure, which was put into the cell plate, and the ECAR and OCR levels were detected. The probe plate was corrected, and the ECAR and OCR were detected.

The morphology of mitochondria as observed by transmission electron microscope.

Cells at the logarithmic phase were digested and centrifuged, and 50 µl cells were precipitated in a new centrifuge tube. After fixing cells in 2.5 % glutaraldehyde for 4 h, the fixed solution was absorbed, and the precipitation was washed with phosphoric acid buffer 3 times. Then the cellular precipitation was fixed with 1% osmic acid for 2 h and then washed with phosphoric acid buffer 3 times. Ethanol gradient dehydration method was selected for dehydration. The sample was put in a 520% acetonitrile solution, then switched into 70%, 80%, 90%, 95% and 100% acetonitrile solution successively (soaking for 15-20 min every time). Finally, it was put in 100% acetonitrile and then dried in a vacuum. The samples were sprayed with carbon and then gold, and the structures of cells and mitochondria were observed under transmission electron microscope.

Label-free quantitative proteomics

The cells were collected, and cellular precipitation was more than 50 µl. The harvested precipitates were suspended in a 8 M urea solution and then got sonicated for 5 minutes. Pyrolysis at 14000×g and 4 °C for 15 minutes to remove any insoluble

substances. Protein concentration of the solution was determined by a BCA protein quantitation method. The solution containing 50 μg protein was disulfide reduced with 5 mM tris - (2-chloroethyl) - phosphoric acid (TCEP) (room temperature, 30 minutes) and alkylated with 10 mm IAA (room temperature, 30 minutes in the dark). Then trichloroacetic acid (TCA) was added to precipitate the protein. The precipitated protein was separated by centrifugation at 14000 g for 30 min, and the particles were washed three times in acetone to remove residual TCA. The dried protein was then reconstituted in 50 mM Tris-HCl (pH 8.2) containing 8 M urea solution. The solution was diluted to a targeted urea concentration of 2 M by 50 mM Tris HCl, and the protein was digested at a 1 / 50 (w / w) enzyme-protein ratio at 37 °C overnight. The same amount of peptide samples was first cleaned with C18 column (based on the monolithic technology of Merck KGaA company in Darmstadt, Germany). Then the obtained peptide sample was loaded onto the column. The liquid passing through the column was labeled as FT, which contained peptides that did not bind or weakly bound to the C18 beads. The column was washed with 300 μl ddH_2O, eluted with 200 μl methanol, and vacuum-dried for the LC-MS/MS analysis. Each sample was separated by HPLC liquid phase system Easy nLC with nanoliter flow rate. The samples were separated by chromatography and analyzed by Q-Exactive mass spectrometer. Data analysis: the original data of mass spectrometry analysis was a raw file, and MaxQuant software was used for database identification and quantitative analysis.

Western blot

The expressions of PDK1, P-AKT and P-GSK3β proteins were detected by Western blot. Cell lysate (RIPA: protease inhibitor: phosphorylated protease inhibitor = 100:2:1) was added to the cell dish to extract the all proteins in the cell. The protein was quantified by the Coomassie brilliant blue protein quantitation method. An appropriate separation gel was selected according to the molecular weight. 40 μg of protein sample was added per lane, and the samples were separated by SDS-PAGE gel

electrophoresis. Then the protein was transferred to the NC membrane. 5% milk or 5% BSA was used for blocking for one hour. Anti-PDK1, P-AKT, P-GSKβ and β-catenin were diluted with antibody diluents at 1000:1 and incubated at 4 ℃ overnight. The next day, after washing the NC membrane, secondary antibody was incubated, and the scanning quantitative analysis was carried out on an Odyssey infrared fluorescence scanning imaging system.

Lactate measurement

We seeded 3×10^5 ishikawa cells in six-well plates for 24 h and then replaced the medium with 3 mL of fresh medium with different treatments. After treatments for 24h, cell supernatant was collected, and then lactate production of cells was measured by infinite M200 multifunctional enzyme-labeling instrument according to the instructions of a Lacate Assay Kit (Njjcbio, A019-2-1).

***In vivo* study**

BALb/c nude mice, 5-week of age, were purchased from the animal laboratory of the People's Hospital of Peking University (Beijing, China) and raised under SPF conditions. The animal experiment was approved by the ethics committee of Peking University People's hospital (2020PHE093), and the ethical requirements of experimental animals and the animal welfare law were strictly adhered to during the experimental operations. BALB/ c nude mice were divided into two major groups, including a diabetic group ($Mice^{Dia+}$) and a non-diabetic group ($Mice^{Dia-}$), First, nude mice in the $Mice^{Dia+}$ group received intraperitoneal injection of streptozotocin (1% w/V solution in fresh cold sodium citrate buffer, pH 4.5) and high fat feed. Nude mice in the $Mice^{Dia-}$ group were given the same volume of citric acid buffer and normal feed. The blood glucose level of mice was detected by blood sugar device. When any random blood glucose concentration went over 11.1mmol/l, the modeling was considered successfully. Ishikawa cell line, of 100 μl which roughly included 3×10^6 cells, was inoculated subcutaneously in nude mice, then ishikawa bearing BALB/c

mice with diabetes (mice$^{EC+/dia+}$) and non-diabetes (mice$^{EC+/dia-}$) were constructed.

The Ishikawa cells in Mice$^{Dia+/sh-NC}$ group were transfected with a PDK1 negative control vector, and the cells in the Mice$^{Dia+/sh-PDK1}$ group were transfected with shRNA-PDK1. When the tumor volume reached about 500 mm^2, the mice$^{EC+/dia-}$ in saline group were treated with saline (n=5) and in Met group were treated with Met dissolved in drinking water (500 mg/L) daily (n=5). The mice$^{EC+/dia+}$ in saline group were treated with saline (n=5) and in Met group were treated with Met dissolved in drinking water (500 mg/L) daily (n=5). The mice$^{EC+/dia-}$ in JX06 group were injected with JX06 1.5 mg/kg (n=5), in JX06-NPs group were injected with JX06-NPs (equivalent to 1.5 mg/kg JX06) (n=5), in JX06+Met group were treated with Met and JX06 1.5 mg/kg, and in JX06-NPs+Met group were treated with Met and JX06-NPs (equivalent to 1.5 mg/kg JX06). The tumor size was measured every 3 days, and the formula used for the calculation of tumor volume was $v = a \times b^2 \times 0.52$, in which a represents the long diameter of the tumor and b represents the short diameter of the tumor. After the experiment, nude mice were killed, and the subcutaneous tumors were removed, photographed, and recorded. In addition, tissue samples were fixed, paraffin-embedded and sectioned for follow-up studies.

Immunohistochemical staining

Immunohistochemical staining was performed on EC tissue arrays, purchased from Superbiotek, Shanghai, including 118 EC tissue samples (patient^{EC+}) and 17 normal control endometrial tissue samples adjacent to EC specimens (patient^{NC}). Immunohistochemical staining was also performed on the tumor tissues from mice$^{EC+/dia+}$ after treatments. The paraffin sections were dewaxed. High temperature and pressure repair method was used for antigen repair. Antigen repair solution (pH6.0 citric acid) was added to the pressure cooker and boiled at a high temperature. The slices were soaked in 3% H_2O_2 for 30 min, removing endogenous peroxidase, and were washed by PBS for 5min for 3 times. Rabbit anti-PDK1 antibody (1:50) or rabbit anti-PDHA1 (Phospho S293) (1:400) was added to the section and put into the

wet box at 4 ℃ overnight. The next day, after rewarming for 30min, it was again rinsed with PBS for 5 min, which was repeated 3 times. HRP-labeled Goat anti-rabbit IgG was added to the sections and incubated at room temperature for 30 min and 3 times washed with PBS for 5 min. DAB solution was added to the tissue, and the color was observed under the microscope. The slides were immersed in water to stop further staining. The slides were counterstained with hematoxylin and differentiated by hydrochloric acid alcohol solution. The slides were dehydrated with 100% ethanol, sealed, observed, scanned, and photographed under a microscope. The results were analyzed by the Fromowitz comprehensive scoring method ①staining intensity score: 0 for no stain, 1 for light yellow particles significantly standing out from the background, 2 for light brown-yellow particles, and 3 for a large number of dark brown particles; ② Score of positive cells: the number of positive cells in 500 cells randomly counted in each piece, < 5% is 0, 5% ~ 25% is 1, 26% ~ 50% is 2, 51% ~ 75% is 3, and > 75% is 4. The sum of the score for staining intensity and the proportion of positive cells was calculated and graded: < 2 points was considered negative (-), 2 ~ 3 points was considered weakly positive (+), 4 ~ 5 points was moderately positive (+), 6 ~ 7 points were highly positive (+ + +), and + ~ + + + were the positive range. According to the immunohistochemistry score, samples were divided into a highly positive PDK1 expression group (high), a moderately positive group (moderate) and a negative or poorly positive group (neg/poor). The EC arrays contained 135 samples. Further, the samples were divided into a group of normal control tissue adjacent to tumor specimen (patients^{NC}, n=17), EC tissue (patients^{EC+}, n=118), non-diabetic EC tissue (patients$^{EC+/dia-}$, n=116), and diabetic EC tissue (patients$^{EC+/dia+}$, n=19), and the expressions of PDK1 in all groups were compared.

Collection of human clinical data

This study collected the clinical data on 506 patients with EC admitted to the Department of Gynecology of Peking University People's Hospital from January 2012 to December 2016, which included medical history, fasting blood glucose levels and

clinicopathological characteristics. Exclusion criteria: Patient with a family history of tumor, a history of chronic kidney disease, a history of other tumors, and those without sufficient clinical data. Patients were divided into two groups based their serum glucose levels or medical history: a normal glucose group (blood glucose level less than 6.1, NG-patient) and a hyperglycemic group (fasting blood glucose more than 6.1 mmol/L or had been diagnosed with diabetes mellitus and currently undergoing diabetic treatment, HG-patient). The study was approved by the ethics committee of the Peking University People's Hospital. The baseline data of patients were shown in Table S1.

Apoptosis detection by flow cytometry

The cells were inoculated into a six-well plate. According to the experimental grouping, the Ishikawa^{HG} were treated with Met 5 mM, JX06(0.5 μM), JX06-NPs (0.5 μM), Met + JX06, and Met + JX06 NPs for 24 to 48 h, respectively. Then, the cells were harvested and washed twice with precooled PBS, centrifuged at 1000 rpm for 3 min, and resuspended with 1x binding buffer with a concentration of 1 x 10^{6} cells / ml. 100 μl (including 1 x 10^{5} cells) of binding buffer was added into the 1ml Ep tube. Subsequently, 5 μl FITC annexin V and 5μL propidium iodide (PI) were added into the Ep tube. The cells were incubated at room temperature (25 °C) for 15 min in a dark room. Then, 400 μl of 1x binding buffer was added to each tube. Finally, the apoptosis analysis was quantified by flow cytometry.

CCK8 to detect cell proliferation

Ishikawa^{NG} or Ishikawa^{HG} were inoculated into 96-well plates, with 100μl per well containing 3000 cells. After an overnight culture, they were transferred to fresh mediums containing specific concentrations of Met or JX06 or rotenone and cultured for 24~72 hours. Then 10 μl of CCK-8 reagent was added to each well, and the plate was incubated at 37 °C for 2 h . Then we detected the absorbance at 450nm with the infinite M200 multifunctional enzyme labeling instrument.

TCGA database and Bioinformatics analysis

We downloaded the mRNA expression profile of endometrial cancer, including 552 EC tissues and 35 normal endometrial tissue samples from TCGA database (https://portal.gdc.cancer.gov/), and obtained the corresponding clinical information on those patients, including tumor grade and survival time. PDK1 expression was extracted from the mRNA expression profile. Wilcox. Test was used to compare the levels of PDK1 between 552 EC tissues and 35 normal tissues. The levels of PDK1 in patients with different grades of tumor (grade, G1, G2 and G3) were compared. Based on the median value of PDK1 expression, patients with type I EC were divided into a PDK1 high expression group and a PDK1 low expression group. The difference in 10-year survival between the two groups was further analyzed by Kaplan Meier survival analysis.

Gene Set Enrichment Analysis，GSEA

GSEA software was used to analyze the signaling pathways with either high or low levels of PDK1 expression. The PDK1 expression file (.gct) and phenotype classification file (.cls) were prepared and were run on the GSEA software. It is generally considered that gene sets with absolute values of NES ≥ 1.0, NOM p-val ≤ 0.05, and FDR q-val ≤ 0.25 are significant.

Synthesis of Polymer1

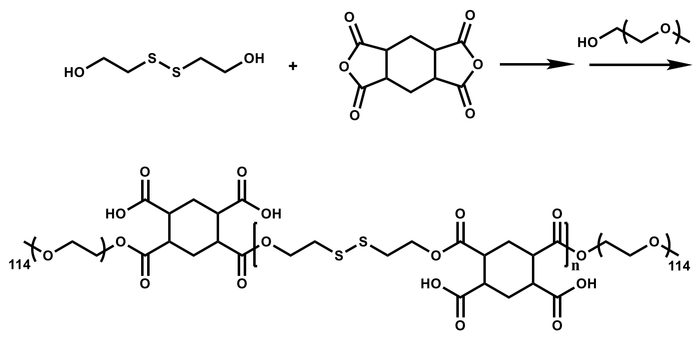

DSB (0.1 mM) and 1,2,4,5-Cyclohexanetetracarboxylic Dianhydride (HPMDA) (0.11 mM) were placed in a 50 mL round-bottom flask, then 10 mL DMF was added into the flask with continuous stirring for 48 h. Subsequently, $mPEG_{5k}$-OH (0.02 mmol) was added to end-cap the polymer for 24 h. The final product poly (DSB-co-HPMDA)-PEG (P1) was collected via dialysis and then dried under a vacuum.

Preparation and characterization of nanoparticles (JX06-NPs)

The as-synthesized polymer P1 (10 mg) and JX06 (1 mg) were initially dissolved in 1 mL of DMSO, and then the solution was dispersed in 10 mL of de-ionized water (43 ℃) with continuous stirring. After vigorous stirring for 15 min, the mixture was collected and dialyzed using a dialysis bag (MWCO: 3500 Da) overnight. The nanoparticles (termed JX06-NPs hereafter) were then separated by centrifugation and washed twice with de-ionized water. The morphology of JX06-NPs was characterized by TEM (HT-7700, Hitachi, Japan). The size of JX06-NPs was measured by a Malvern Zetasizer Nano ZS90 laser particle size analyzer (Nano ZS, UK).

Drug release kinetics of nanoparticles

The in vitro drug release kinetics study of nanoparticles was carried out by dialysis using PBS (pH = 7.4) or an aqueous solution (GSH = 10 mM) as the release medium. Seal 2 mL of nanoparticles in a dialysis bag (molecular retention of 3000), and then immerse them in 100 mL of release medium in a beaker covered with aluminum foil. Keep the beaker at 37°C while stirring at 100 rpm. At different time points, 1 mL of the solution was taken from the dialysate and measured by HPLC.

Drug Combination Index

A combination index (CI) values were obtained to analyze the efficacy in inhibiting cell proliferation between JX06 and Met by using Compusyn software. The CI value

correlates with the effect of combination treatment. A CI of <0.9 is considered synergistic, a CI of $\geq$ 0.9 or $\leq$1.1 is considered additive, and a CI of >1.1 is considered antagonistic [42]. The cell proliferation was deteched by CCK-8 experiment, and the result was analyzed after 48h treatment.

In Vivo Imaging and Biodistribution Analysis

Ishikawa cells (3×10^6) were subcutaneously injected into the hip of female BALB/c mice. When the tumor volumes reached about 1000 mm^3, mice were i.v. injected with JX06-NPs@Cy7.5. After injection, the fluorescence signals were recorded on IVIS spectrum imaging system (Spectrum CT, PerkinElmer) at 1, 6, 12, 24, 36, 48 h. For the biodistribution study, mice were sacrificed after 48 h post injection, and the tumors and normal organs were harvested and imaged.

Statistical Analysis

Data were presented as mean ± standard deviation (SD). The data was analysis on GraphPadPrism 8. Student's t test, one-way ANOVA, Kaplan-Meier method and Chi-square tests were performed. Bioinformatics analysis was performed on R software. Differences were considered statistically significant at a level of $*p < 0.05$; $**p < 0.01$;

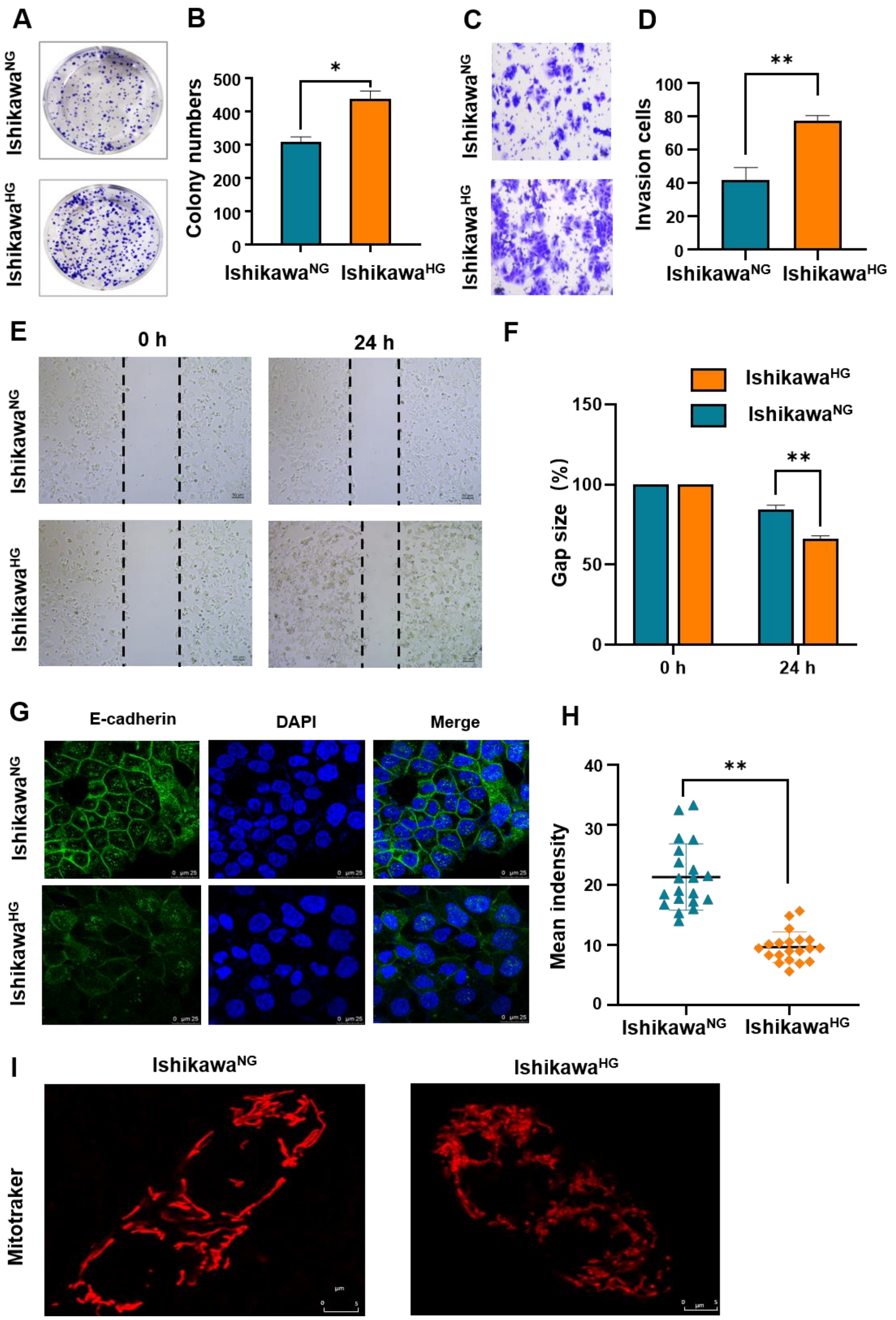

Figure S1. High glucose promoted not only the proliferation, invasion, and migration, but also EMT of endometrial cancer cells. (A) The colony-forming experiment was used to detect the clones formed in the Ishikawa^{NG} and Ishikawa^{HG}. (B) The statistical analysis. (C) The effect of glucose on the invasiveness of Ishikawa

endometrial cancer cells was detected by Transwell analysis. (D) Statistical analysis on the numbers of cells passing through Transwell ventricular membrane either in a high glucose medium or in a normal glucose. (E) Wound healing assay was used to measure the area of the healing zone of $Ishikawa^{NG}$ and $Ishikawa^{HG}$ at the start of the experiment and after 24 hours. (F) The statistical analysis. (G) The expressions of E-cadherin on the surface of $Ishikawa^{NG}$ and $Ishikawa^{HG}$ were detected by immunofluorescence assay. (H) Statistical analysis of the intensity of E-cadherin-marking fluorescence expression in $Ishikawa^{NG}$ and $Ishikawa^{HG}$ (20 cells were counted). (I) The effect of high glucose on mitochondrial morphology as detected by confocal microscopy. Mitochondria were labeled by mitotracker, and the mitochondria in $Ishikawa^{NG}$ and $Ishikawa^{HG}$ were under confocal microscope. Data are shown as mean ± SD. Unpaired Student's t test. $*P < 0.05$, $**P < 0.01$.

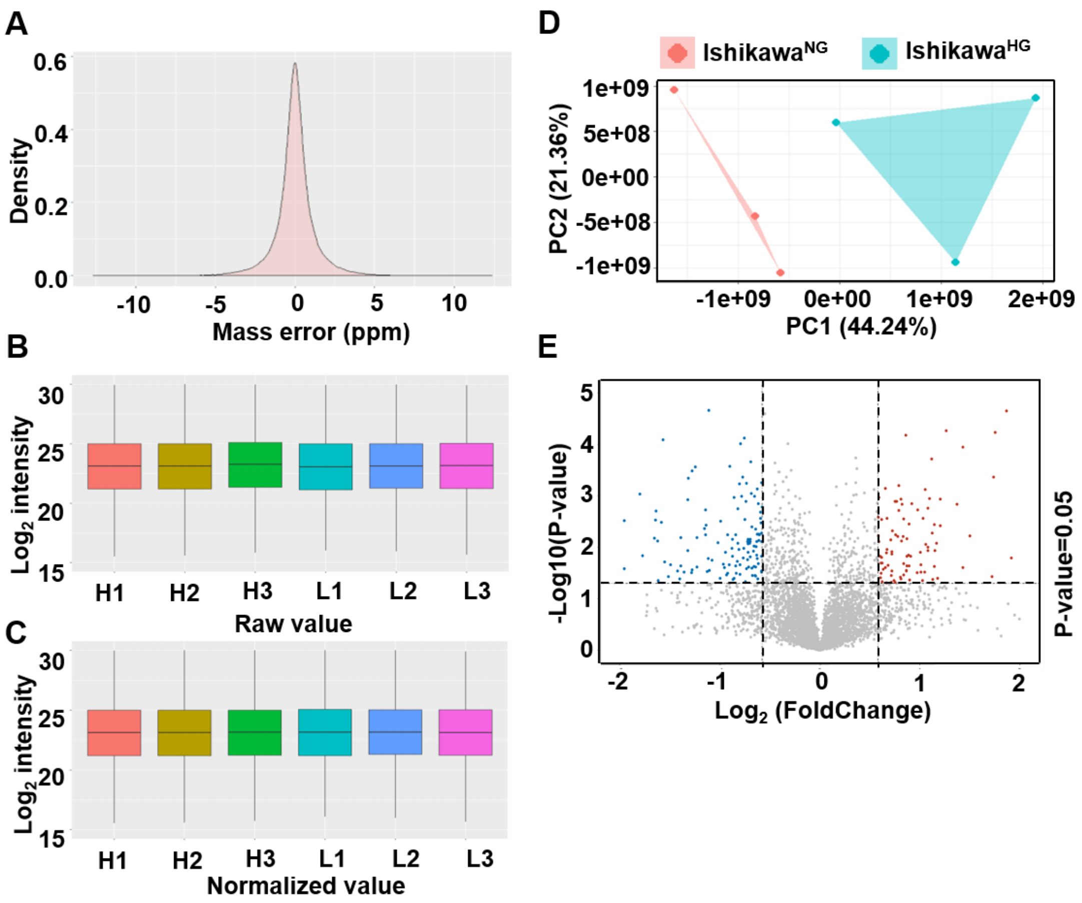

Figure S2. Screening differentially expressed proteins by mass spectrometry proteomics. (A) The distribution of errors in the mass of all identified peptides. Errors in the mass of the identified peptides were basically within 2.5 ppm, exemplifying the precision of the mass spectrometry method. (B) Raw data. (C) Standardized data. (D) The different distributions of expressed proteins between the high glucose group and the normal glucose group were analyzed by Principal Components Analysis (PCA). Different colors represented different groups, and the coordinate axis reflected two components of the sample difference (PC1 and PC2); The scale on the coordinate axis was a relative distance; The percentage represented the relative contribution of each component. (E) Volcano diagram of differentially expressed proteins. By comparison, red dots are proteins with significantly upregulated expression, blue dots are downregulated proteins, and gray dots are proteins with levels no different between the two groups.

A

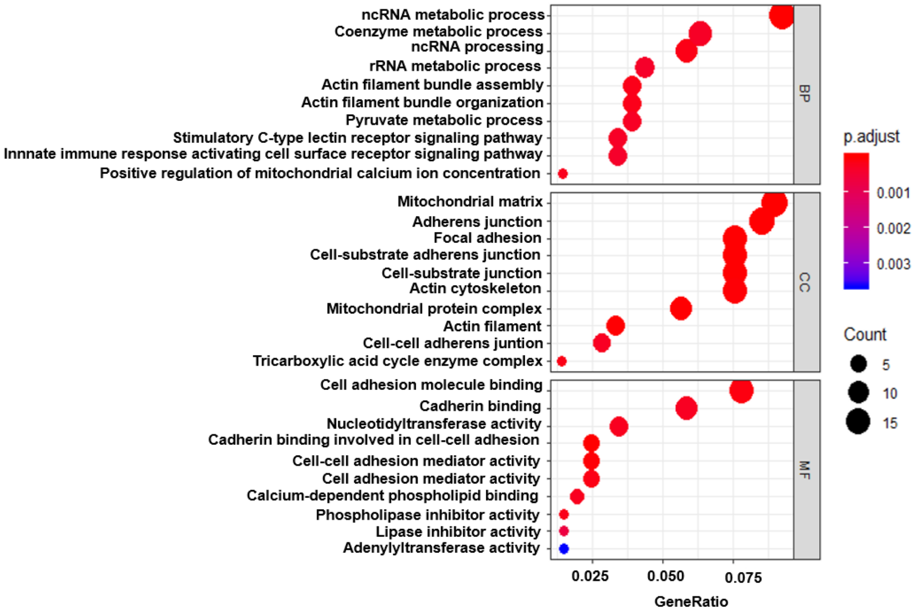

B

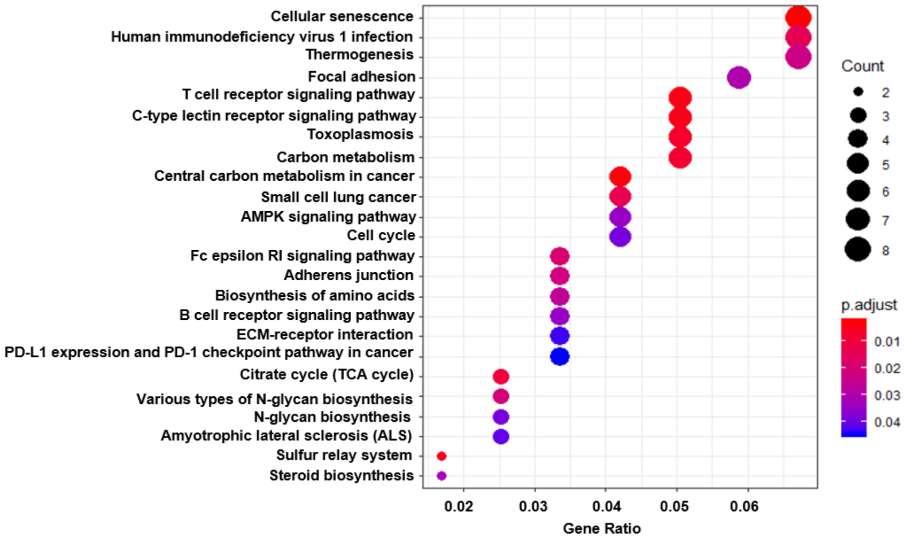

Figure S3. GO and KEGG enrichment analysis of differential protein expressions when comparing those under high glucose and normal glucose conditions. (A) GO enrichment analysis of differential protein expression. GO mainly included BP, CC and MF. The condition for screening was a p.adjust < 0.05. The size of bubbles represented the number of differentially expressed proteins, and the color represented the degrees of these differences, and the GeneRatio was the ratio of the number of proteins related to this term to the total number of differential proteins. (B) KEGG enrichment analysis of differentially expressed proteins. Bubble size

represented the number of differentially expressed proteins, and color represented the degrees of these differences.

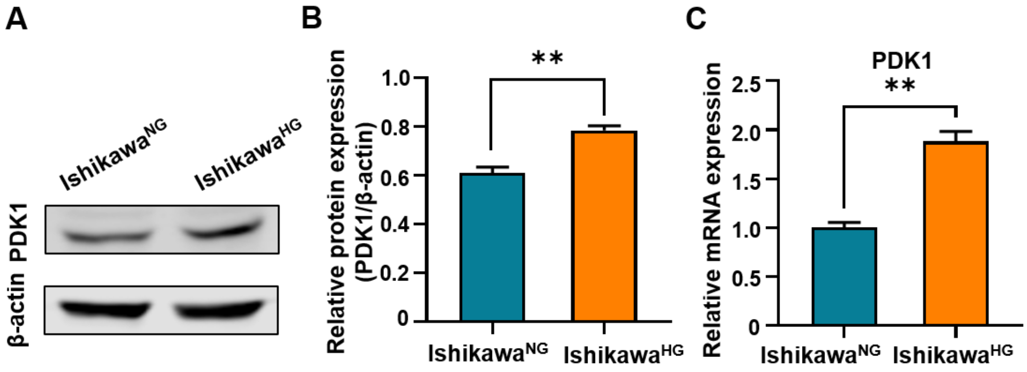

Figure S4. The effect of high glucose on PDK1 expression at protein and mRNA levels. (A,B) PDK1 expressions in Ishikawa^{NG} and Ishikawa^{HG} groups were detected by western blot. (C) RT-PCR was used to detect the expression of PDK1 mRNA in Ishikawa^{NG} and Ishikawa^{HG}. Results were from a representative experiment in triplicates. Data are shown as mean ± SD. Unpaired Student's t test. ***P* < 0.01.

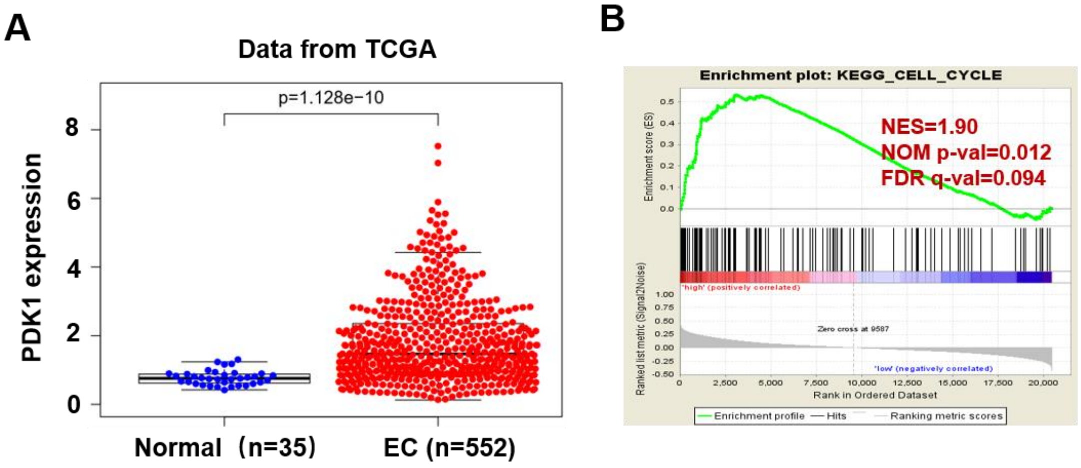

Figure S5. GSEA enrichment analysis and the expressions of PDK1 in EC and adjacent normal tissues. (A) PDK1 expressions in 552 cases of EC and 552 cases of normal tissues from the TCGA database. (B) GSEA analyzed the significant signaling pathways enriched with PDK1. Data are shown as mean ± SD. (A) Wilcox.test by R (3.6.3) .

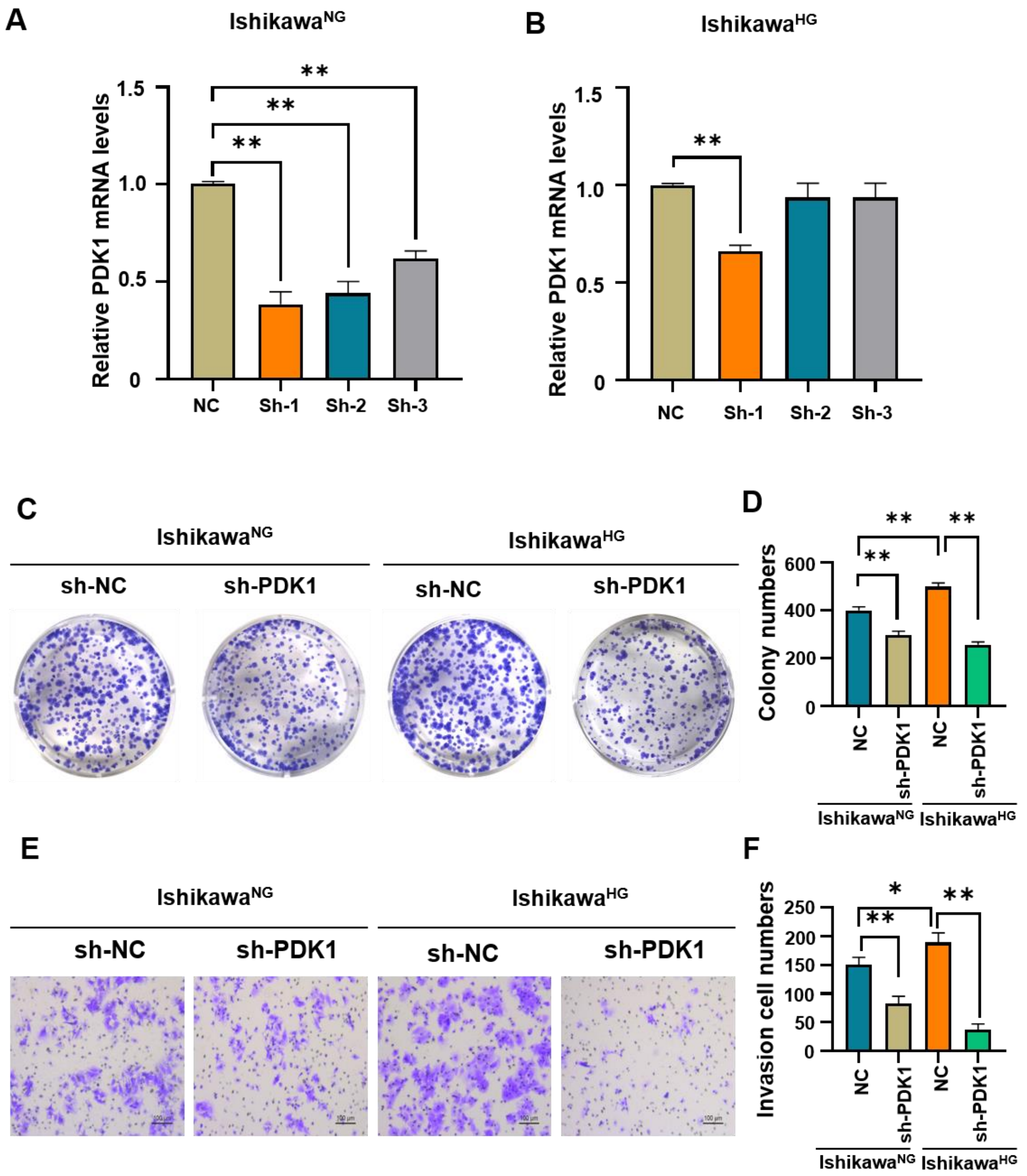

Figure S6. Downregulate the expression of PDK1 by shRNA could significantly inhibit the proliferation and invasion of endometrial cancer cells. (A,B) To verify the downregulatory effect of PDK1 on Ishikawa^{NG} and Ishikawa^{HG} after transfected with sh-NC, shRNA-1 (sh-1) , shRNA-2 (sh-2), and the shRNA-3 (sh-3). (C-D) Clone formation assay was used to detect the effect of PDK1 regulation on the proliferation of Ishikawa EC cells. (E-F) Transwell assay was used to detect the effect of PDK1 knockdown on the invasive behavior of Ishikawa EC cells. Data are shown as mean ± SD. One-way ANOVA. * $P < 0.05$, ** $P < 0.01$.

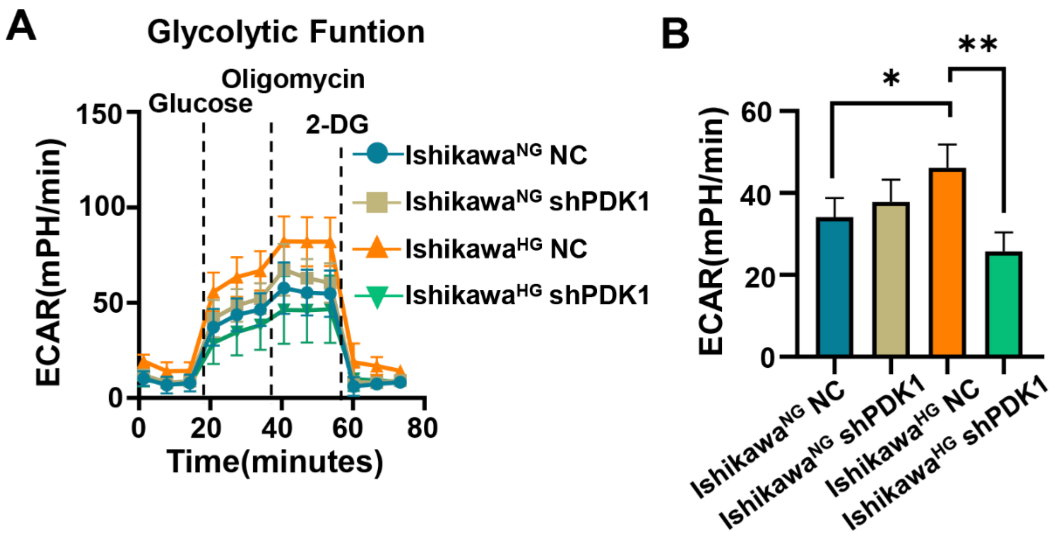

Figure S7. Seahorse cell energy metabolism analysis was used to detect the effect of PDK1 downregulation on the ECAR of Ishikawa EC cells. The average ECARs of Ishikawa^{NG} and Ishikawa^{HG} of NC group and sh-PDK1 group were compared. The glycolytic capacity of cells was detected after adding glucose, and the maximum glycolytic capacity of cells was detected after the addition of oligomycin, After the addition of 2-DG, the mechanisms of acid production other than glycolysis was detected. Data are shown as mean ± SD. One-way ANOVA. * $P < 0.05$, ** $P < 0.01$.

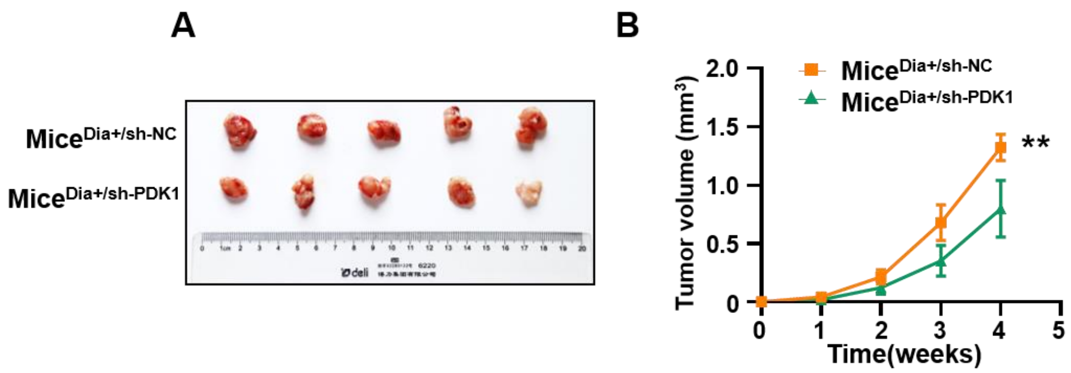

Figure S8. To verify the *in vivo* effect of PDK1 downregulation on tumor growth in Ishikawa-bearing diabetic mice. (A) Pictures of the tumor tissues from mice$^{Dia+/sh\text{-}NC}$ group and the mice$^{Dia+/sh\text{-}PDK1}$ group (n=5). (B) The tumor volumes in the mice$^{Dia+/sh\text{-}NC}$ group and the mice$^{Dia+/sh\text{-}PDK1}$ group were recorded, and the growth curve was drawn. mice$^{Dia+/sh\text{-}PDK1}$ vs mice$^{Dia+/sh\text{-}NC}$, Data are shown as mean ± SD. Unpaired Student's t test. $^{**}P < 0.01$.

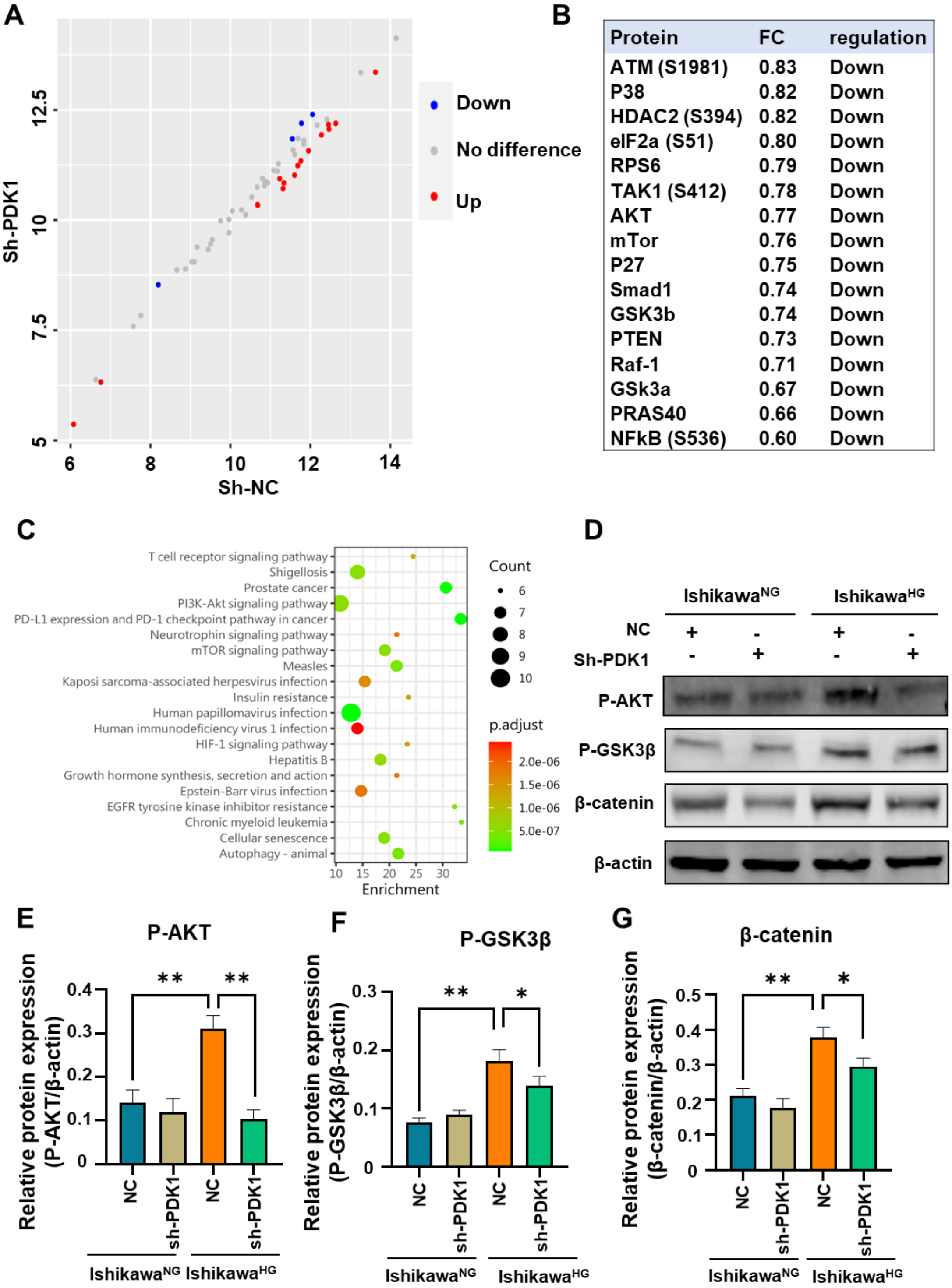

Figure S9. Screening of molecular signaling pathways downstream of PDK1 by phosphorylated antibody chip. (A) Differentially expressed proteins of Ishikawa^{HG} in the sh-NC and sh-PDK1 groups. Screening conditions: fold change ≥ 1.2, up: the

protein was upregulated in the sh-NC group relative to that in the sh-PDK1 group; Down: downregulated protein in the sh-NC group when compared to its counterpart in the sh-PDK1 group; No difference: proteins with no significant difference between the two groups. (B) The downregulated proteins in sh-PDK1 group when compared to those in sh-NC group. (C) The differentially expressed proteins were analyzed by KEGG enrichment. p.adjust < 0.05, The circle represented the number of proteins. (D) Western blot was used to detect the expressions of P-Akt, P-GSK3β, and β-catenin in $Ishikawa^{NG}$ and $Ishikawa^{HG}$ of NC group and sh-PDK1 group, respectively. (E-G) The relative expressions of P-AKT, P-GSK3β and β-catenin in $Ishikawa^{NG}$ and $Ishikawa^{HG}$ of NC group and sh-PDK1 group, respectively. Data are shown as mean ± SD. One-way ANOVA. * $P < 0.05$, ** $P < 0.01$.

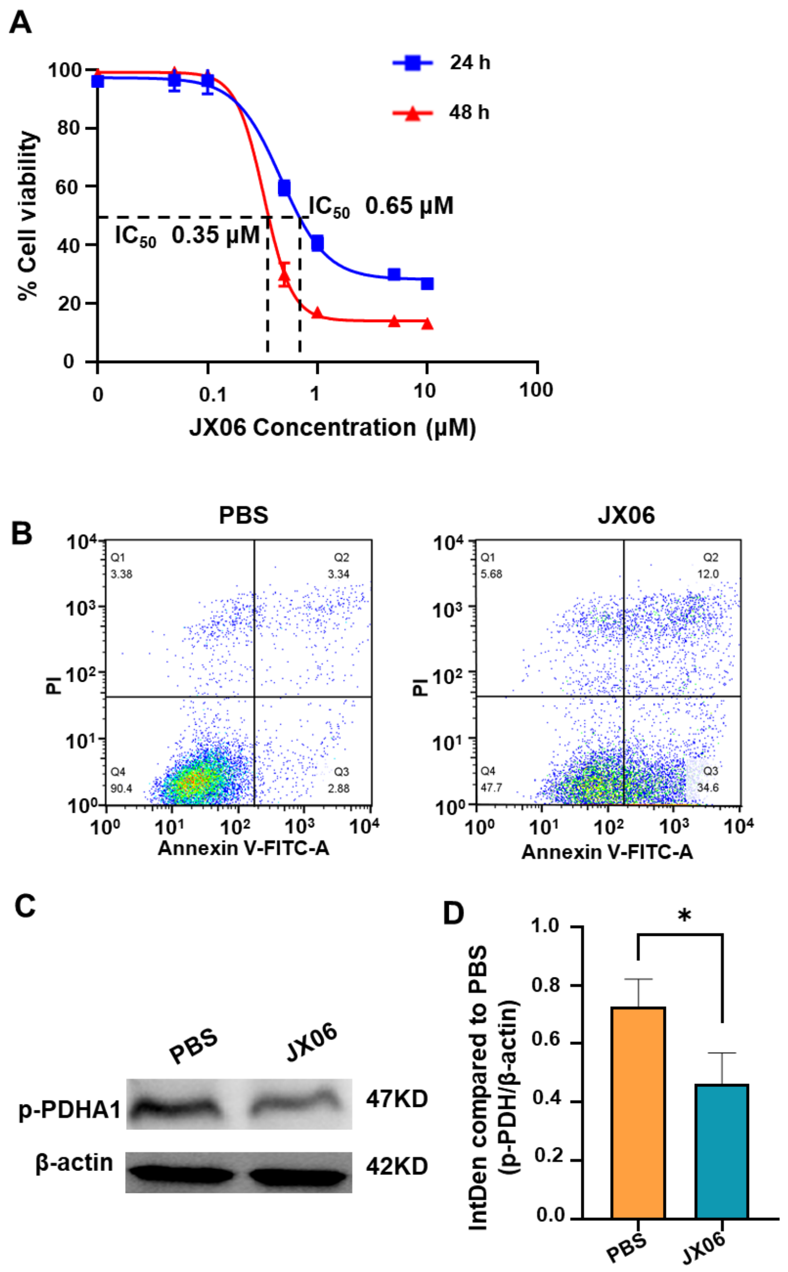

Figure S10. JX06 inhibited the proliferation of endometrial cancer cells and also promoted their apoptosis. (A) CCK-8 assay was used to detect the effect of JX06 on the proliferation of Ishikawa^{HG}. (B) The effect of JX06 on apoptosis of Ishikawa^{HG} was detected by flow cytometry. (C, D) Western blot was used to detect the expression of p-PDHA1 in Ishikawa^{HG} treated with JX06. Results of Western blot were from a representative experiment in triplicates. Data are shown as mean ± SD. Unpaired

Student’s t test. *P < 0.05.

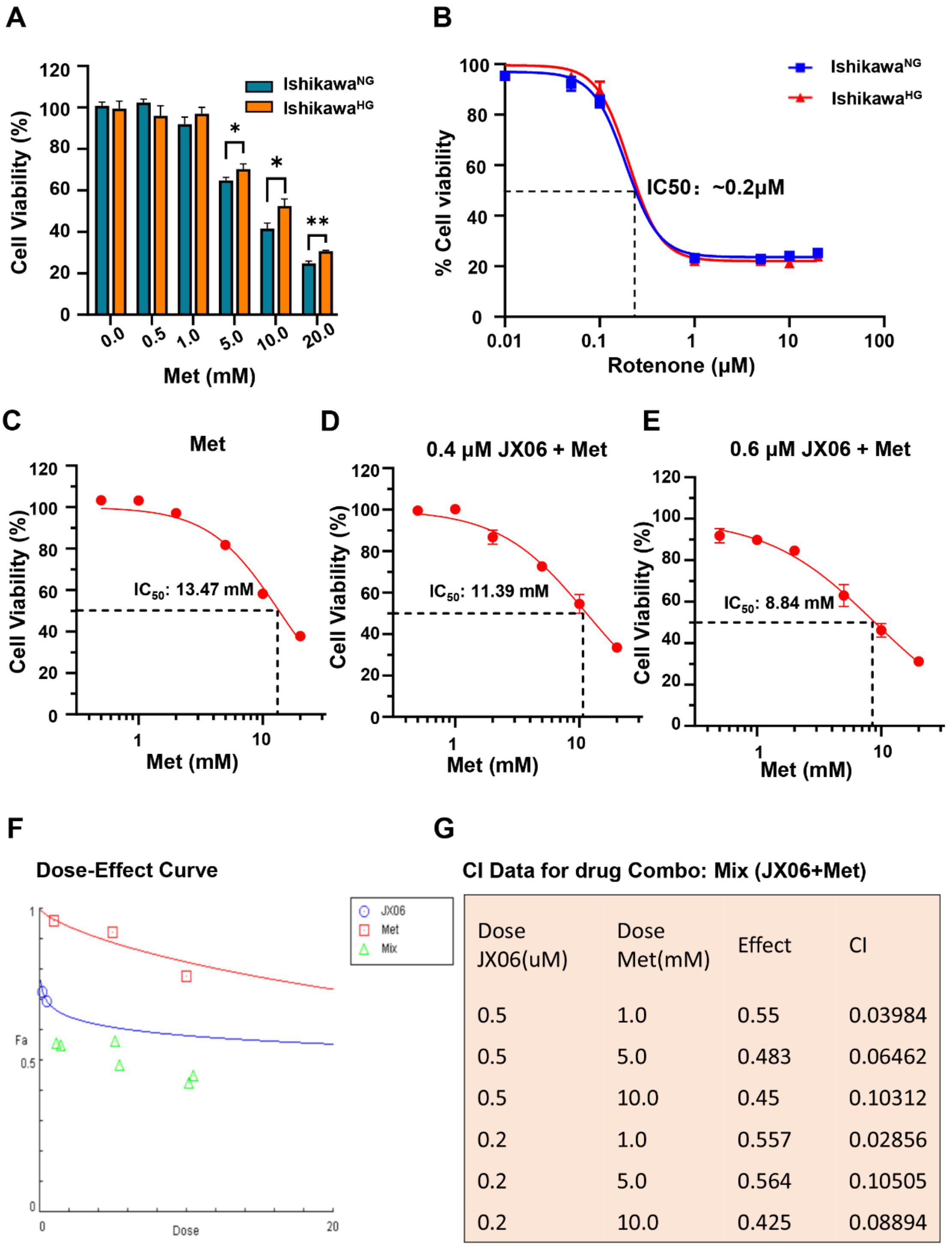

Figure S11. Effect of JX06 combined with Met on the proliferation of Ishikawa EC cells. (A) Effects of different concentrations of Met on the proliferation of Ishikawa^{NG} and Ishikawa^{HG}. (B) The effect of rotenone on the proliferation of Ishikawa^{NG} and Ishikawa^{HG}. (C-E) The IC_{50} of Met on Ishikawa^{HG} treated with Met

alone, Met+0.4 μM JX06, and Met + 0.6 μM JX06. (F) Dose-effect curve of JX06, Met and mix (Met+JX06) were generated by using compuSyn software. Fa: Fraction affected. (G) The CI data for mix (JX06+Met) was calculated by using compuSyn software. Data are shown as mean ± SD. Unpaired Student's t test. **P* < 0.05. ***P* < 0.01.

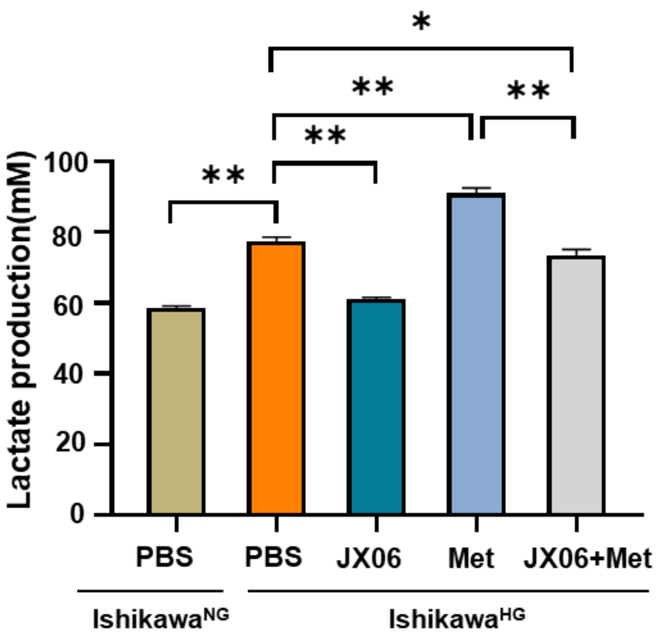

Figure S12. Effect of JX06 combined with Met on the lactate production of Ishikawa EC cells. Data are shown as mean ± SD. Unpaired Student's t test and One-way ANOVA. **P* < 0.05. ***P* < 0.01.

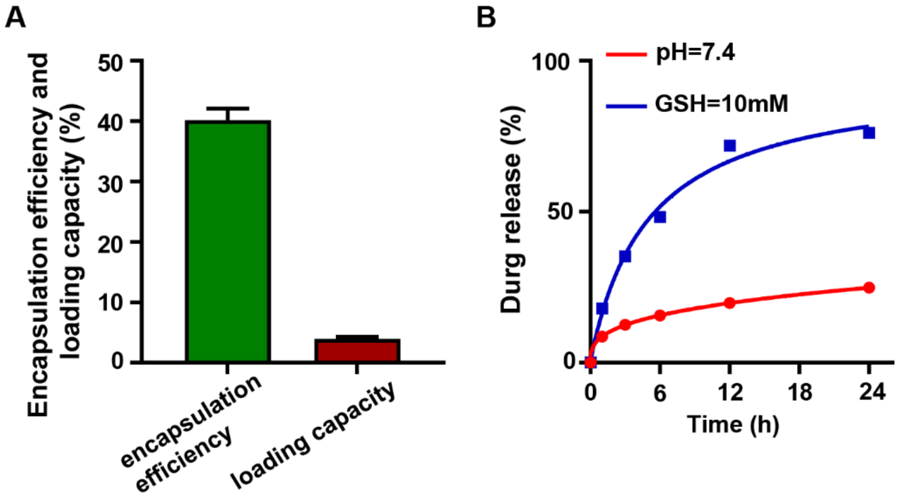

Figure S13. JX06 encapsulation efficiency, loading capacity and drug release. (A) JX06 encapsulation efficiency and loading capacity (%). (B) Drug (JX06) release at pH 7.4 and GSH 10mM from 0 h to 24 h.

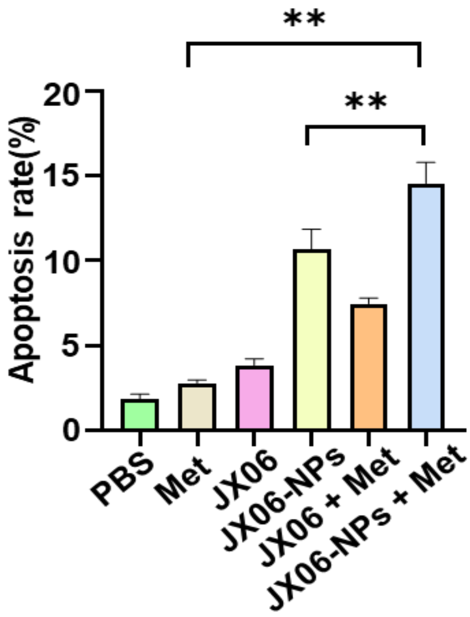

Figure S14. Effects of different treatments on apoptosis of Ishikawa^{HG}. The cell apoptosis rate was determined by Annexin-V/PI double staining and flow cytometry. Statistical graphics of apoptosis rate under different treatments. Data are shown as mean ± SD. One-way ANOVA. $^{**}P < 0.01$.

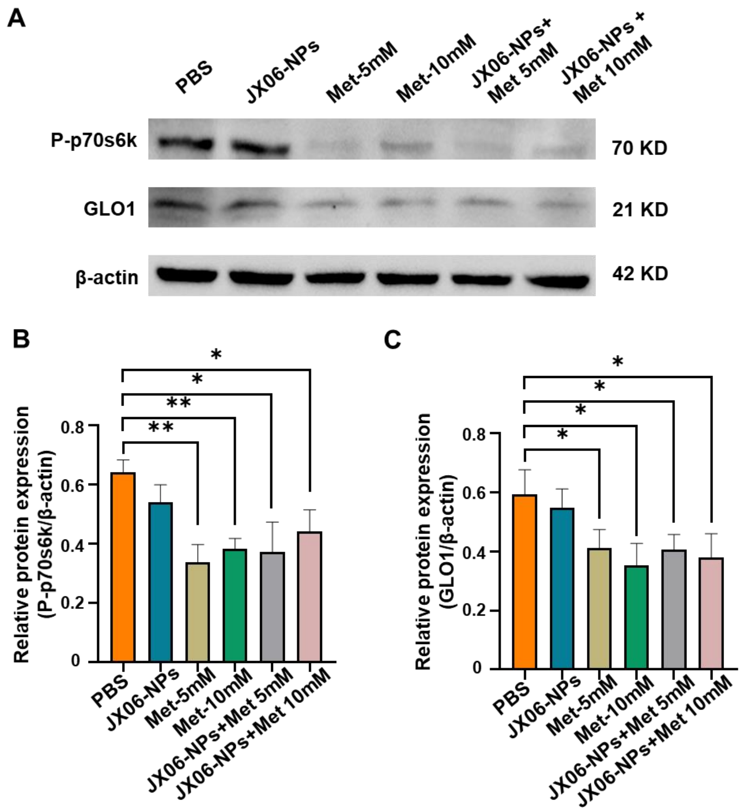

Figure S15. The effect of single and combinational use of metformin and JX06-NPs on the expression of P-p70s6k and GLO1. (A) The protein gray of western blot. (B-C) The relative protein expression of P-p70s6k and GLO1, respectively. Data are shown as mean ± SD. One-way ANOVA. * $P < 0.05$, ** $P < 0.01$.

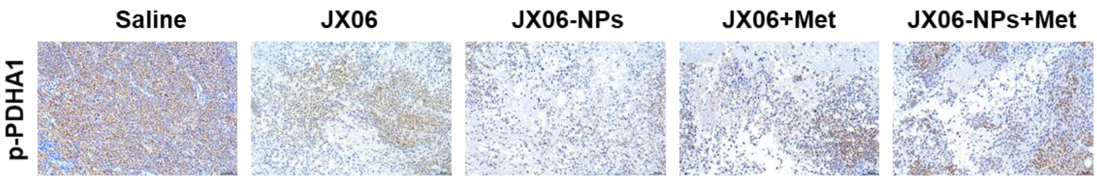

Figure S16. The expression of p-PDHA1 in tumor tissue from mice$^{EC+/dia+}$ was detected after treatments by immunohistochemistry. Brown granules represent positive expression (×200). Scale bar: 50μm.

Table S1. Baseline characteristics of 506 EC patients (n, %)

variable	Glucose level	
	Normal glucose (n=305)	High glucose (n=201)
age	54.00(49.00,60.00)	58.00(52.00,63.00)
Tumor grade		
Grade1	235(77.05)	152(75.62)
Grade 2-3	70(22.95)	49(24.38)
FIGO stage		
Stage I	248(81.31)	154(76.62)
Stage II-IV	57(18.69)	47(23.38)
Ascites tumor cells		
Negative	207(89.61)	145(92.95)
Positive	24(10.39)	11(7.05)
Lymph node metastasis		
Negative	224(88.54)	140(83.83)
Positive	29(11.46)	27(16.17)
LVSI		
Negative	261(85.57)	158(78.61)
Positive	44(14.43)	43(21.39)
MI		
MI-superficial	215(70.49)	122(60.70)
MI-deep	90(29.51)	79(39.30)

Table S2. The top ten up-regulated differential proteins in Ishikawa^{HG} compared with Ishikawa^{NG}

ID	Symbol	Fold Change (H/L)	P-value
Q02952	AKA12	5.96	0.00
P16070	CD44	4.92	0.00
Q9NZI8	IGF2BP1	4.20	0.00
O43490	CD133	3.76	0.02
Q15417	CNN3	3.64	0.00
Q9UHJ6	SHPK	3.36	0.00
Q15118	PDK1	3.33	0.00
P60903	S10AA	3.29	0.04
O15392	BIRC5	2.82	0.01
P52298	NCBP2	2.69	0.02

、

Table S3. The shRNA sequences of PDK1

Name	Sequence
PDK1-NC-F	GATCCGTTCTCCGAACGTGTCACGTAATTCAAGAGATTACG TGACACGTTCGGAGAATTTTTTC
PDK1-NC-R	AATTGAAAAAATTCTCCGAACGTGTCACGTAATCTCTTGAA TTACGTGACACGTTCGGAGAACG
PDK1-sh1-F	GATCCGCATGACCCTGTGGATGGGAGAATTATTCAAGAGA TAATTCTCCCATCCACAGGGTCATGTTTTTTG
PDK1-sh1-R	AATTCAAAAAACATGACCCTGTGGATGGGAGAATTATCTC TTGAATAATTCTCCCATCCACAGGGTCATGCG
PDK1-sh2-F	GATCCGCAGACCACGTGTGTGATGTGGTGTTTTCAAGAGA AACACCACATCACACACGTGGTCTGTTTTTTG
PDK1-sh2-R	AATTCAAAAAACAGACCACGTGTGTGATGTGGTGTTTCTCT TGAAAACACCACATCACACACGTGGTCTGCG
PDK1-sh3-F	GATCCGGCCATGTGGAAATGTGCACAGGAATTCAAGAGAT TCCTGTGCACATTTCCACATGGCCTTTTTTG
PDK1-sh3-R	AATTCAAAAAAGGCCATGTGGAAATGTGCACAGGAATCTC TTGAATTCCTGTGCACATTTCCACATGGCCG

Annotation

**Name**	**Code**
EC patients with diabetes	$patient^{EC+/dia+}$
Ishikawa cells cultured in normal glucose medium	$Ishikawa^{NG}$
Ishikawa cells cultured in normal glucose medium	$Ishikawa^{HG}$
EC bearing diabetic mice	$mice^{EC+/dia+}$
EC tissue samples	$patient^{EC+}$
Adjacent normal control endometrial tissue samples	$patient^{NC}$
EC patients with non-diabetic	$patient^{EC+/dia-}$
A diabetic BALB/c nude mice model	$Mice^{Dia+}$
$Ishikawa^{HG}$ transfected with sh-NC were injected subcutaneously to the $Mice^{Dia+}$	$Mice^{Dia+/sh-NC}$
$Ishikawa^{HG}$ transfected with sh-PDK1 were injected subcutaneously to the $Mice^{Dia+}$	$Mice^{Dia+/sh-PDK1}$
